# The supragenic organization of glycoside hydrolase encoding genes reveals distinct strategies for carbohydrate utilization in bacteria

**DOI:** 10.3389/fmicb.2023.1179206

**Published:** 2023-06-02

**Authors:** Renaud Berlemont

**Affiliations:** Department of Biological Sciences, California State University – Long Beach, Long Beach, CA, United States

**Keywords:** glycoside hydrolase, cellulase, CAZy, genomics, polysaccharide utilization, gene cluster, sugar transporter

## Abstract

Glycoside hydrolases (GHs) are carbohydrate-active enzymes essential for many environmental (e.g., carbon cycling) and biotechnological (e.g., biofuels) processes. The complete processing of carbohydrates by bacteria requires many enzymes acting synergistically. Here, I investigated the clustered or scattered distribution of 406,337 GH-genes and their association with transporter genes identified in 15,640 completely sequenced bacterial genomes. Different bacterial lineages displayed conserved levels of clustered or scattered GH-genes, but overall, the GH-genes clustering was generally higher than in randomized genomes. In lineages with highly clustered GH-genes (e.g., *Bacteroides, Paenibacillus*), clustered genes shared the same orientation. These codirectional gene clusters potentially facilitate the genes' co-expression by allowing transcriptional read-through and, at least in some cases, forming operons. In several taxa, the GH-genes clustered with distinct types of transporter genes. The type of transporter genes and the distribution of the so-called GH:TR-genes clusters were conserved in selected lineages. Globally, the phylogenetically conserved clustering of the GH-genes with transporter genes highlights the central function of carbohydrate processing across bacterial lineages. In addition, in bacteria with the most identified GH-genes, the genomic adaptations for carbohydrate processing also mirrored the broad environmental origin of the sequenced strains (e.g., soil and mammal gut) suggesting that a combination of evolutionary history and the environment selects for the specific supragenic organization of the GH-genes supporting the carbohydrate processing in bacterial genomes.

## 1. Introduction

Bacteria are essential for the processing of carbohydrates across ecosystems and thus contribute greatly to global carbon cycling (Berlemont and Martiny, 2016; López-Mondéjar et al., 2022). Although both autotrophic and heterotrophic bacteria have evolved ways to process endogenous carbohydrates (e.g., starch/glycogen), some heterotrophs, frequently referred to as the “carbohydrate degraders,” have evolved many enzymes to degrade the polysaccharides in their environment (Talamantes et al., 2016; Berlemont, 2017; Terrapon et al., 2018).

The complete deconstruction of carbohydrates requires many interacting carbohydrate-active enzymes (CAZymes). For example, regarding the cellulose, some extracellular enzymes cleave the polysaccharides, such as cellulases from the glycoside hydrolase family 6 (GH6), and release short oligosaccharides (e.g., cellobiose) (Berlemont and Martiny, 2013; Nguyen et al., 2018). Then, these products can be further processed outside or inside the cell, after translocation, by specific ‘osidases' such as the GH3 β-glucosidases releasing monosaccharides (Berlemont and Martiny, 2013). Inside the cell, monosaccharides are then channeled through glycolysis or other catabolic and anabolic pathways. Bacteria and fungi with a high potential for carbohydrate deconstruction are associated with a large repertoire of CAZymes. This includes many single-domain GHs sometimes associated with accessory non-catalytic domains (e.g., carbohydrate-binding modules—CBMs) (Talamantes et al., 2016; Nguyen et al., 2018), complex non-covalent multi-proteins assemblies combining several catalytic domains acting synergistically (i.e., cellulosome) (Ravachol et al., 2014; Artzi et al., 2016), and a few multi-activity proteins (MAPs) with several catalytic domains (Brunecky et al., 2013; Zhang et al., 2014; Kim et al., 2017; Nguyen et al., 2019).

CAZymes, and their corresponding gene, can be identified in sequenced genomes datasets and the functional potential for carbohydrate processing of a sequenced organism correspond to its predicted ability to process polysaccharides based on the repertoire of identified CAZymes (Berlemont and Martiny, 2015). As the processing of carbohydrates requires multiple activities, the corresponding genes are often co-regulated (Amore et al., 2013; Tani et al., 2014). Genes in a “regulon” (i.e., sets of genes that share the same regulation) have similar cis-regulatory elements or share the same cis-regulatory element in operons. The identification of regulons in sequenced microbial genomes is not trivial (Hiard et al., 2007; Bouyioukos et al., 2016) since transcription factors are often poorly conserved across lineages, thus limiting the regulon prediction at large (Madan Babu et al., 2006; Junier and Rivoire, 2016). However, besides well-characterized regulons, genome-wide analyses revealed that the co-expression of independent genes is affected by their physical distance, expressed in nucleotides, or sometimes as the number of genes (Pál and Hurst, 2004). Compared to genes located in distant regions of the chromosome, closely located genes tend to be co-expressed (Junier and Rivoire, 2016). Thus, beyond tightly regulated supragenic structures (e.g., operon), the physical clustering of genes encoding functionally coupled proteins (i.e., functional coupling) fosters their co-expression, potentially improving the fitness of their host, and thus might be selected under specific environmental conditions (Fang et al., 2008). In this context, clusters of independent genes might represent an evolutionary intermediate between scattered genes and operons (Junier and Rivoire, 2016).

As the complete deconstruction of carbohydrates requires multiple functionally coupled enzymes, the corresponding genes are predicted to be clustered. Interestingly, the identification of “polysaccharide utilization loci” (PULs) in members of the *Bacteroides* highlighted the clustered distribution of GH-genes targeting polysaccharides (Terrapon et al., 2018). In PULs, many of the predicted GH-genes and other CAZymes were associated with sugar transporters (Larsbrink et al., 2014) together forming some clustered GH-genes with transporter genes (GH:TR-cluster). In *Bacteroides*, these sugar transporters, referred to as SusCD or SUS transporters, are generally TonB-dependent transporter (TBDT, SusC) that interact with SusD lipoproteins although some TBDTs are involved in the uptake of other nutrients (Pollet et al., 2021). TBDTs are multi-domain proteins with a central barrel domain (PF00593) in the outer membranes connected to a “plug” domain (PF07715) in periplasmic space. In *Bacteroides*, additional domains such as the N-terminal extension domain (NTE, PF13715), also identified as DUF4480 domain (PF13715) and, in some cases, a short N-terminal domain (STN, PF07660), are sometimes associated with the “core” TBDT. Although the functional coupling of GHs and Sus-transporters in some PULs has been biochemically confirmed (e.g., Larsbrink et al., 2014; McKee et al., 2021), most PULs were predicted bioinformatically (Terrapon et al., 2015, 2018; Ausland et al., 2021). In addition, PULs identified in Bacteroidetes are the CAZyme gene clusters (CGCs) consisting of “physically linked genes clusters that encode at least one CAZyme, one transporter, one transcriptional regulator, and one signaling transduction protein” (Zhang et al., 2018). Some CGCs have been predicted in lineages other than Bacteroidetes, mainly using a curated literature search (Ausland et al., 2021).

The completely sequenced *Bacteroides* genomes contain the highest proportion of GH-genes (up to ~7.5% of the PEGs) but PULs have been predicted in other Bacteroidetes, where both the number of identified GHs and the frequency of GHs within PULs vary extensively. According to the PulDB, none of the 10 predicted GHs in *Alistipes putredinis* DSM17216 were in a PUL, whereas 35% of 119 predicted GHs from *Tannerella sp*. were in PULs. Finally, 51% of the 399 predicted GHs (out of 5,244 PEGs) in *B. cellulolysiticus* DSM14838 are located in PULs (Terrapon et al., 2018). This raises some questions about the genomic features supporting the carbohydrate deconstruction (e.g., clustering of the GH-genes) in *Bacteroides* genomes and in other lineages.

First, besides functional coupling, what are the factors affecting the physical clustering of GH-genes in bacterial genomes? I predicted that the amount (or the frequency) of GH-genes would be a determining factor supporting the formation of clusters. Although having more genes of interest could increase the frequency of observed clusters, it is unclear how these numbers change with varying overall genome size and the number of GH-genes. Second, is the GH-gene clustering (with transporter gene) conserved across bacterial lineages? Just like the distribution of GH-genes is conserved across bacterial genera (Berlemont and Martiny, 2013, 2015), I predicted that the physical distribution (clustered or scattered) of GH-genes would be conserved in discrete bacterial lineages. Finally, I was interested in elucidating the association of GH-genes with the various types of sugar transporters (i.e., ABC, PTS, MFS, and SUS) across completely sequenced bacterial genomes. Since, transporter genes are not evenly distributed across microbial lineages (e.g., McKee et al., 2021; Wang et al., 2022), I predicted that the association between GH- and transporter genes is phylogenetically constrained and mirrored the organism's potential to respond to the carbohydrate supply.

The increasing number of publicly accessible sequenced microbial genomes (Wattam et al., 2017), and the development of the precise annotation system for GHs (e.g., Huang et al., 2018; Nguyen et al., 2018, 2019; Zhang et al., 2018) provided an unprecedented opportunity to investigate the physical clustering of GH-genes across bacteria. Hence, I first generated randomized genomes to investigate how the number of GH-genes and the overall genome size affect the clustering of GH-genes, in the absence of selection. In addition to highlighting the factors affecting the GH-genes clustering in randomized genomes, this simulation provided a baseline to estimate the likelihood that an observed clustering, in an actual genome, is random. Next, I identified the GH-genes in 15,640 completely sequenced and publicly accessible genomes retrieved from the BV-BRC (formerly known as the PATRICbrc) database (Wattam et al., 2017). This data was used to investigate the physical clustering of GH-genes (and transporter genes) across bacterial lineages.

## 2. Materials and methods

### 2.1. Genome randomization

Bacterial genomes with 1,000 to 15,000 protein-encoding genes (PEGs) which contained 0.5% to 10% of GH-genes (from 5 to 750) were simulated in the R statistical environment (Script in Supplementary Data). For each condition, random distributions of 5,000 GH-genes were generated.

The synteny score (SSc), for each GH-gene, was defined as the shortest distance (upstream or downstream) expressed in the number of PEGs, between two successive GH-genes. Then, the number (N_SSc_) and frequency (F_SSc_) of GH-genes with synteny scores ranging from 1 to 20 were obtained for each genome. Finally, for each set of conditions, the normality of N_SSc_ and F_SSc_ was tested using the Shapiro–Wilk test implemented in the R statistical environment.

### 2.2. Gene identification and mapping

In June 2022, I retrieved the protein sequences and the complete taxonomy of 15,640 bacterial genomes from the BV-BRC database, formerly known as the PATRICbrc database (https://www.bv-brc.org) (Wattam et al., 2014, 2017). To avoid missing GH-genes and bias introduced by sequence fragmentation in draft genomes, I focused on the “complete” genomes only (i.e., genomes with no ambiguous bases and in which the contigs equal the number of chromosomes). The genes of interest encoded protein with at least one glycoside hydrolase domain (GHs, Supplementary Table 1) and transporter domains (TRs, Supplementary Table 2). The precise identification of the genes of interest was achieved using GeneHunt (Talamantes et al., 2016; Nguyen et al., 2019) and HMM-profiles retrieved from the Pfam database (35.0, Supplementary Tables 1, 2) (Finn et al., 2014). A tailored version of GeneHunt designed to mine completely sequenced genomes from the BV-BRC database is available at https://doi.org/10.6084/m9.figshare.22207552.v1. In addition, the “protein encoding gene” (PEG) number and the orientation of the coding strand, provided by the BV-BRC database, were used as a proxy to identify the position and orientation of the genes of interest. Next, the SSc was computed for each pair of sequential GH-genes, in all the genomes, as described for the simulation data.

### 2.3. Data processing

All the data analytics, statistics, and visualization were done using the R environment with the vegan (v2.5-6), ggplot2 (v3.3.2), dplyr (v1.0.1), reshape2 (v1.4.3), and splitstackshape (v1.4.8).

## 3. Results

### 3.1. Bacterial genome randomization

In order to elucidate how the genome size (expressed as the number of protein-encoding genes—PEGs) and the number of the genes of interest, in this case, GH-genes affected the clustering of these genes, I simulated GH-genes distributions in randomized bacterial genomes (Supplementary Figure 1). First, when the GH-genes accounted for < 1% of the PEGs, the average distance between successive GH-genes (average synteny score—SSc) was highly variable and not normally distributed (Shapiro–Wilk test, *p* < 0.01 and W < 0.98). Conversely, when the frequency of GH-genes increased, the average SSc decreased and approached the normal distribution (Shapiro–Wilk, *p* > 0.01 and W > 0.98) (Supplementary Figure 1A). Next, when focusing on random bacterial genomes with 5,000 PEGs (Supplementary Figure 1B), the number of GH-genes with low SSc (i.e., more clustered distribution) increased non-linearly with the number of GH-genes. Thus, in genomes with few genes of interest, most of the GH-genes are expected to be scattered. Next, as the number of GH-genes increased, the frequency of clustered genes increased as well. For example, in a purely random genome with 5,000 PEGs and 300 GH-genes (6%), one should expect to identify up to ~35 clustered GH-genes (i.e., SSc = 1). However, this number increased quickly, not linearly, when also considering separated GH-genes (i.e., SSc > 1) (Supplementary Figure 1B). Thus, both the number of GH-genes and the size of the genomes, as well as the synteny score used to define the clusters affect the expected number of clustered genes. In actual microbial genomes, change in the GH-genes frequency mirror variation in the GH-genes repertoire and the genome size (expressed in PEGs). This suggests that in genomes enriched in GH-genes, such as some *Bacteroides*, the observation of clusters could, at least partially, mirror some stochastic processes resulting from the high frequency of GH-genes.

### 3.2. GH-genes identification and mapping in sequenced bacterial genomes

As of June 2022, the predicted protein sequences from 15,640 “complete” bacterial genomes were retrieved from the BV-BRC database and analyzed using GeneHunt to identify the genes encoding proteins with at least one GH domain.

The dataset was strongly biased toward pathogens and other microbes relevant to human health including 929 *Escherichia*, 864 *Salmonella*, 710 *Streptococcus*, and 697 *Staphylococcus*, among others. This dataset also included 194 *Streptomyces*, 29 *Bacteroides*, and a few other well-known polysaccharide degraders including 9 *Caldicellulosiruptor*, 7 *Cellulomonas*, and 5 *Ruminococcus*, among others. Finally, many taxa were associated with only a few “complete” genomes.

In total, GeneHunt identified 406,337 proteins for a total of 754,960 protein domains (Supplementary Data 1). Although 202,258 proteins consisted of single-domain GHs (SDGHs), 204,079 were multi-domain proteins (MDGHs) with some proteins containing more than 10 domains. For example, fig|113107.26.peg.548 from *Streptococcus australis* strain NCTC5338, encoded a GH2 (i.e., β-galactosidase) with 15 predicted discrete protein domains.

Across genomes, the most abundant GH domains identified were 55,091 predicted GH13 α-amylases (PF00128), 32,825 predicted GH1 β-glucosidases (PF00232), and 27,813 predicted GH3 β-glucosidases (PF00933). Domains for enzymes targeting structural polysaccharides such as cellulose and xylan were less abundant. Specifically, the most abundant GH domains endowed with predicted cellulolytic activity were 8,276 GH5 (PF00150), 5,572 GH8 (PF01270), and 1,695 GH6 (PF01341), whereas 2,677 GH10 (PF00331) and 703 GH11 (PF00457) encoding putative xylanases were identified. Regarding domains for predicted chitinases, 13,745 and 2,893 domains for GH18 (PF00704) and GH19 (PF00182) were identified. Regarding the proteins with multiple domains, many non-GH domains including CBMs, dockerin domain, and some domains of unknown function (DUFs), among others, were found associated with the GH domains (Supplementary Data 1).

**Figure 1:**
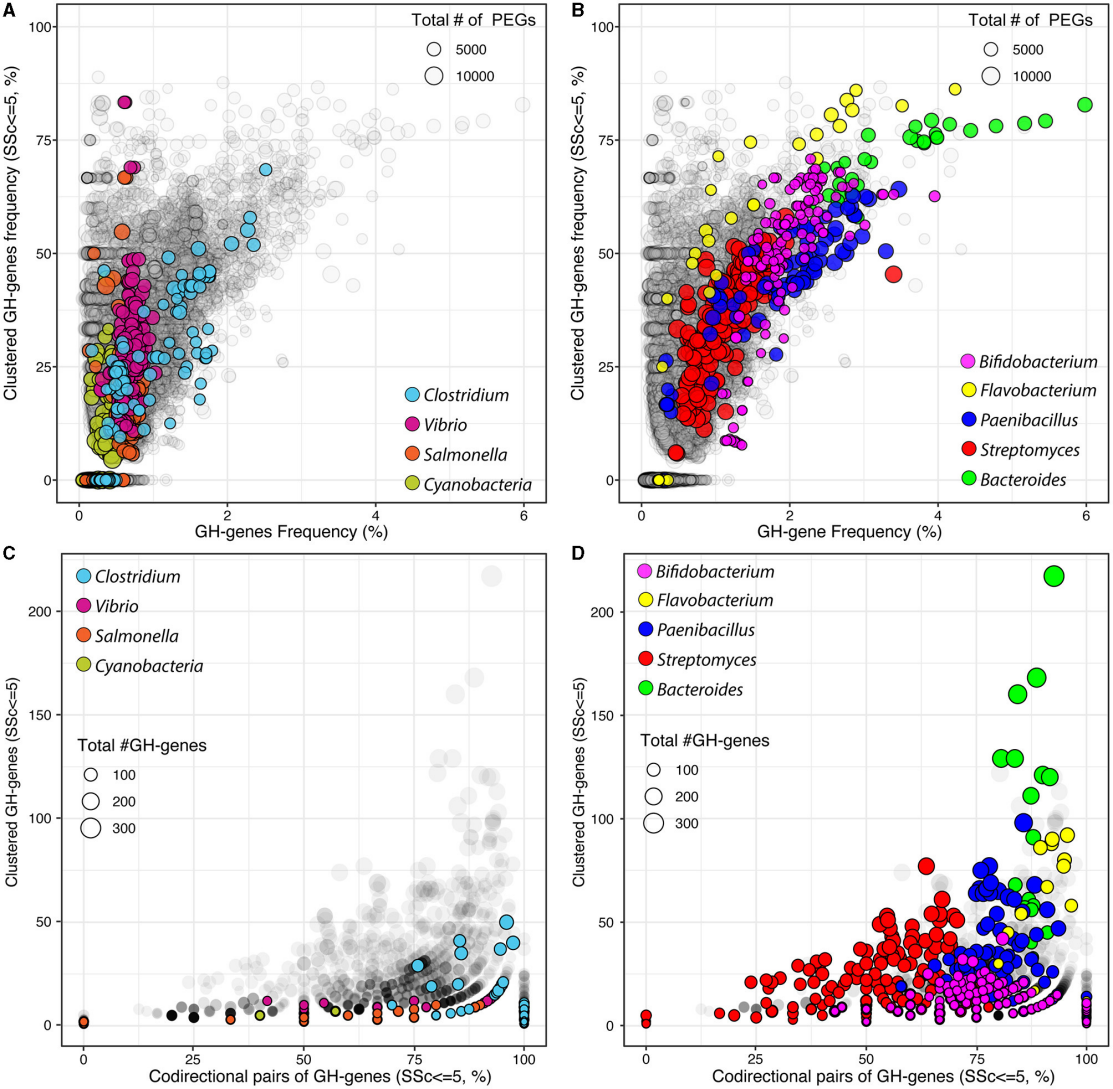
Frequency of GH-genes and clustered GH-genes (SSc ≤ 5) in completely sequenced bacterial genomes (gray) and in selected lineages with **(A)** reduced or **(B)** high potential for carbohydrate utilization. Frequency of codirectional genes in the identified GH-gene clusters (SSc ≤ 5) in completely sequenced bacterial genomes (gray) and in selected lineages with **(C)** reduced or **(D)** high potential for carbohydrate utilization.

Across genomes, the average frequency of GH-genes was low (0.6%) but ranged from ~0.03% to ~6% of the PEGs (Figure 1). Overall, the number of identified GH-genes correlated with the genome size (r_Pearson_ = 0.38, *p* < 0.001 and slope = 0.005, *p* < 0.001, Table 1). Although conserved in many genera (e.g., *Streptomyces* and *Bacteroides*), this trend was not systematic. For example, among the Actinobacteria, variation of the genome size was strongly associated with the number of GH-genes in *Clavibacter, Cutibacterium*, and *Streptomyces*, whereas in Bacteroidetes, variation in the genome size of *Bacteroides, Flavobacterium*, and *Hymenobacter* mirrored the GH-gene content. In the Firmicutes phylum, *Leuconostoc* and *Paenibacillus* were the lineages with the highest correlation between the number of GH-genes and the overall genome size. Finally, in proteobacteria, the genome size of most genera weakly reflected the variation in the GH-gene content (Table 1 and Supplementary Table 3).

**Table 1:** Relationship between the total number of PEGs and the number of identified GH-genes in selected microbial lineages using Pearson's correlation and linear regression.

	**Pearson (*r*)**	**Slope**	**% Variance explained by**			
			**#PEGs**	**#GHs**	**#PEGs x #GHs**	**Res**.
Eubacteria (15,378)	0.377^***^	0.005	< 0.01^*^	1.9^***^	(NS)	98.1
Actinobacterium (1,700)	0.663^***^	0.011	23.6^***^	58.8^***^	(NS)	17.6
*Bifidobacterium* (146)	0.447^***^	0.02	16.6^***^	61.1^***^	1.0^*^	21.3
*Streptomyces* (199)	0.622^***^	0.018	19.3^***^	40.7^***^	(NS)	39.9
Bacteroidetes (489)	0.649^***^	0.029	27.8^***^	67.7^***^	(NS)	4.4
*Bacteroides* (30)	0.792^***^	0.071	57.5^***^	41.9^***^	(NS)	0.6
*Flavobacterium* (40)	0.845^***^	0.049	65.6^***^	33.9^***^	0.1^**^	0.4
Cyanobacteria (169)	0.871^***^	0.004	24.4^***^	21.7^***^	(NS)	53.8
Firmicutes (3,884)	0.381^***^	0.007	5.8^***^	74.7^***^	0.0^**^	19.4
*Clostridium* (127)	0.459^***^	0.015	17.2^***^	42.2^***^	(NS)	40.5
*Paenibacillus* (79)	0.683^***^	0.043	39.5^***^	56.0^***^	0.3^*^	4.2

Percentages of the estimated variation in GH-gene clustering (SSc ≤ 5) explained by the total number of PEGs, a proxy for the genome size, the number of GH-genes, and their interaction. Estimates were derived from the ANOVA model (GH-genes_SSc ≤ 5_~PEGs × GH-genes). Significance levels ^***^P < 0.001 < ^**^P < 0.01 < ^*^P < 0.05 < non-significant (NS). For complete statistics of all bacterial genera with ≥10 completely sequenced genomes, see Supplementary Table 3.

**Figure 2:**
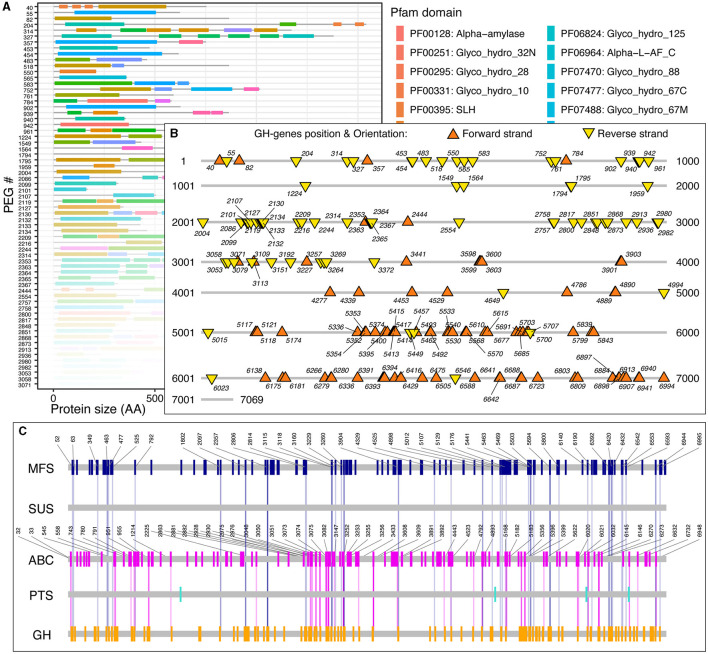
*Paenibacillus cellulositrophicus* strain KACC 16577 (7,069 PEGs) analysis. **(A)** Domain-specific identification of the GH-proteins (partial). The gene ids correspond to the PEG number as provided by the BV-BRC database. Similar figures were created for the MFS, ABC, PTS, and SUS transporters. **(B)** Localization and orientation of the identified GH-genes on the completely sequenced genome from *P. cellulositrophicus* strain KACC 16577. Numbers, when displayed, correspond to the PEG number. **(C)** Transporter identification and GH-gene colocalization. The vertical lines connecting the GH-genes (orange) and the transporter genes highlight GH:TC clusters with SSc ≤ 10. Numbers, when displayed, correspond to the PEG number. Similar figures, in high resolution, detailed statistics, and sequences, for 15,640 fully sequenced bacterial genomes are publicly available at https://figshare.com/account/home#/projects/161209.

Next, for each GH-genes in each genome, I identified the SSc and created graphical representations of the corresponding genome to highlight the protein-domain architecture (Figure 2A), as well as their localization and orientation (i.e., forward vs. reverse strand, Figure 2B). The provided data also contain the domain-specific identification of transporter proteins and genes and their co-localization with GH-genes (Figure 2C, discussed below). For example, among the 7,069 predicted protein-encoding genes (PEGs) in *Paenibacillus cellulositrophicus* strain KACC 16577 (Genome ID:562959.3), 160 were predicted to be GH-genes (2.2% of the PEGs, Figure 2). In this bacterium, many GH-genes were scattered over the entire genome. However, although mostly scattered, the orientation of the GH-genes was uneven with the coding DNA strand of most GH-genes in the first half of the chromosome located on the forward strand, whereas GH-genes on the second half of the chromosome were located on the opposite strand (Figure 2B). On the contrary, most of the 179 identified GH-genes (3.7%) in *Bacteroides dorei* strain HS2 L 2 B 045b (4,817 predicted PEGs) appeared in clusters (Supplementary Figure 2).

### 3.3. GH-genes clustering

I next investigated the frequency of clustered GH-genes, defined as the sequential GH-genes with SSc ≤ 5, across 15,640 genomes (Figures 1A, B). This value was selected to identify clusters of closely linked but non-contiguous GH-genes. The clustering of GH-genes was highly variable across genomes and most genomes with no clustered GH-genes, including some *Clostridia, Vibrio*, and *Salmonella* as well as some *Mycoplasma, Burkholderia*, and *Mycobacteria*, were generally small genomes with a reduced number of GH-genes (< 1% of PEGs, Figure 1A). Most autotrophs such as members of the Cyanobacteria phylum (*n* = 153) were also associated with reduced frequency of generally scattered GH-genes. However, several genomes, with reduced potential for polysaccharide utilization, displayed a high proportion (i.e., >50%) of clustered GH-genes (Figure 1A). In these genomes, large variations in the frequency of clustered GH-genes eventually mirrored minor changes in the actual number of clustered GH-genes.

Next, I evaluated the GH-genes clustering within selected bacterial genera with many completely sequenced genomes and/or associated with a high potential for carbohydrate processing (Table 1 and Supplementary Table 3). At large, and as expected, the GH-gene clustering increased with the frequency of GH-genes (Figure 1). In some lineages, such as *Vibrio* and *Salmonella*, the GH-gene clustering varied extensively (from 0 to >75% clustered GH-genes) although the GH-gene frequency displayed little variation (Figure 1A). In these bacteria with few (mostly scattered) GH-genes, the number of GH-genes correlated with the number of PEGs but the actual number of GH-genes and the genome size only marginally explained their clustering (Table 1).

In the phylum Actinobacterium, *Streptomyces* (*n* = 199 genomes), mostly derived from soil, were associated with 33 (*S. cattleya* NRRL 8057) to 282 (*S. chartreusis* NRRL 3882) identified GH-genes. In this genus, the number of GH-genes correlated with the overall genome size (Figure 1B and Table 1). However, as most characterized *Streptomyces* had large genomes ranging from ~5,600 to ~12,300 PEGs, the frequency of GH-genes remained relatively low compared to other lineages with less GH-genes (e.g., *Bifidobacterium*). The majority of analyzed *Streptomyces* displayed mostly scattered GH-genes (average F_SSc ≤ 5_ = 35.2 ± 11.1%, Figure 1B). Yet, in this group, the variation of the GH-gene clustering was significantly explained by both the number of GH-genes (40.7% of the variance) and the genome size (19.3% of the variance, Table 1). Unlike *Streptomyces*, members of the *Bifidobacterium* genus (*n* = 146 genomes), mostly derived from the gastrointestinal tract of mammals, had small genomes ranging from ~1,400 to ~2,750 PEGs associated with 21 to 107 identified GH-genes. Although having less GH-genes than *Streptomyces, Bifidobacteria* contained a higher proportion of these genes and displayed a higher frequency of clustered GH-genes (average F_SSc ≤ 5_ = 49.3 ± 16%, Figure 1B and Table 1). Like *Streptomyces*, the clustering of GH-genes in *Bifidobacterium* was mostly explained by variation in the number of GH-genes.

In *Clostridia* (*n* = 127 genomes, phylum Firmicutes), the number of PEGs, the frequency of GH-genes, and their clustering varied extensively (Figure 1A and Table 1). Specifically, mostly pathogenic strains of *C. tetani* had small genomes (< 3,000 PEGs) with a reduced number of GH-genes (~10) whereas *C. phytofermentans, C. cellulovorans*, and *C. saccharoperbutylacetonicum*, with larger genomes (~5,000 PEGs), were associated with more than 100 GH-genes each. As expected, this disproportionated enrichment in GH-genes (i.e., >10 × ) relative to the overall increase in the total number of PEGs (~1.6 × ) explained the increased number of clustered GH-genes in some *Clostridia* (Figure 1A and Table 1). The same trend was observed in *Paenibacillus* (*n* = 79 genomes) although both the frequency of GH-genes (1.9 ± 0.7%) and the frequency of clustered GH-genes (44 ± 13%) was higher, on average, than in *Clostridium* (Figure 1B).

In the Bacteroidetes phylum, *Bacteroides* (*n* = 30 genomes) and *Flavobacterium* (*n* = 40 genomes) genera were compared. These two groups contain genomes highly enriched in GH-genes and in which the GH-genes content was strongly correlated with the genome size (Figure 1B and Table 1). *Flavobacterium* had variable genome sizes ranging from 2,563 to 4,606 PEGs and containing between 11 and 312 GH-genes. Specifically, 14 *F. psychrophilum* and 2 *F. columnare* with small genomes (~2,500 PEGs) contained very few scattered GH-genes, whereas *F*. sp. PK15 (3,262 PEGs) had 86.2% of 302 identified GH-genes in clusters (Figure 1B). Next, although being also highly variable in their genome size (from ~3,000 to ~6,300 PEGs), the analyzed Bacteroidetes displayed consistently high GH-gene frequency (3.5 ± 0.9%). In both *Bacteroides* and *Flavobacterium*, the variation in the number of clustered GH-genes, although being significantly explained by the number of GH-genes, was also strongly affected by the genome size (Table 1). In most genera, the clustering of GH-genes was best explained by their abundance. However, the overall genome size explained a large fraction of the GH-gene clustering in several lineages including *Cutibacterium* (Actinobacterium), *Bacteroides* (Bacteroidetes), *Lactobacillus* (Firmicutes), and *Cronobacter* (γ-proteobacteria), among others (Table 1 and Supplementary Table 3).

Finally, I investigated the GH-gene codirectional orientation, on the forward or reverse strand. Briefly, in the absence of selection, the odds that two sequential GH-genes share the same orientation are described as a random binomial distribution, and thus 50% of the pair of sequential GH-genes should share the same orientation. When considering all the GH-genes, scattered or clustered, identified in all the genomes, and without accounting for potential bias resulting from uneven lineage distribution, 57.8 ± 17.3% of the sequential pairs of GH-genes shared the same orientation. This value was higher than expected for a perfectly random distribution. Among others, this can be attributed to the gene codirectionality, relative to the origin of replication, observed in some lineages such as *Paenibacillus* (Figure 2B). However, when focusing on the clustered GH-genes (i.e., SSc ≤ 5), 70.0 ± 32.2% of the pairs of sequential GH-genes shared the same orientation thus suggesting that colocalized genes involved in the same process (i.e., carbohydrate processing) tend to form codirectional gene clusters. I next investigated this trend in previously selected bacterial lineages (Figures 1C, D). In *Clostridium* and *Vibrio*, on average, 95.3 ± 11.8% and 89.9 ± 14.6% of the pairs of clustered and sequential GH-genes shared the same orientation, respectively. In *Salmonella*, few identified pairs of clustered GH-genes were colinear (< 50%, Figure 1C). In Cyanobacteria, with few clustered GH-genes, 62.2 ± 42.7% of the clustered and sequential pairs of GH-genes shared the same orientation. In selected lineages with high potential for polysaccharide utilization, most clustered and sequential GH-genes shared the same direction (Figure 1D). More specifically, on average, 76.2 ± 15.4%, 91.7 ± 7.6%, 81.7 ± 9.4%, and 86.9 ± 3.0%, of the clustered GH-genes identified in *Bifidobacterium, Flavobacterium, Paenibacillus*, and *Bacteroides* shared the same orientation, respectively. Interestingly, in *Streptomyces*, only 54.4 ± 17.1% of the clustered and sequential GH-genes identified shared the same orientation (Figure 1D). This suggested that in most lineages, except in *Streptomyces*, there is a strong selection for clustered GH-genes to share the same orientation. Although codirectional gene cluster are a prerequisite to the formation of bacterial operons, it is to be noted that this is also necessary for the transcriptional read-through leading to the co-expression of independent genes.

### 3.4. GH-genes clustering in bacteria with high potential for carbohydrate deconstruction

**Figure 3:**
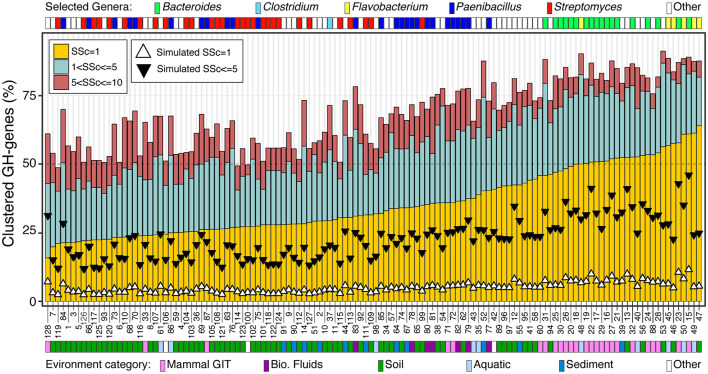
Frequency of GH-gene clusters, with SSc ranging from 1 to 10, in 128 selected bacteria with the highest potential for carbohydrate deconstruction (i.e., >130 identified GH-genes). Triangles represent the median value of 5,000 genome randomizations with the same number of PEGs and GH-genes. For detailed information about the strains, see Supplementary Table 4.

I next focused on the 128 genomes most enriched in GH-genes (i.e., >130 GH-genes) (Figure 3, Supplementary Figure 3, and Supplementary Table 4). The genomes included some *Streptomyces* (*n* = 28), *Paenibacillus* (*n* = 26), and *Bacteroides* (*n* = 18). Some less abundant lineages with high potential for carbohydrate deconstruction were also identified (e.g., *Actinoplanes derwentensis* DSM43941, *Mariniflexile* sp. TRM1-10, and *Paraprevotella xylaniphila* YIT 11841) (Figure 3 and Supplementary Table 4). I manually retrieved the information about the environmental origin of these strains, and the environments were grouped into broad categories. For example, the genome from microbes derived from the human gut, feces, and mouth were all combined in the mammal gastro-intestine tract (i.e., mammal GIT) category (Figure 3 and Supplementary Table 4). The genomes with more scattered GH-genes were generally large genomes and were associated with a stable number of GH-genes, whereas the genomes with more clustered GH-genes had smaller genomes associated with a large but variable repertoire of GH-genes (Figure 3 and Supplementary Figure 3). In these genomes, the frequency of contiguous GH-genes (SSc = 1) was the most variable and ranged from < 20% to more than 60% of the GH-genes. The frequency of GH-genes with 1 < SSc ≤ 5 and 5 < SSc ≤ 10 displayed reduced variation (Figure 3). Interestingly, all the *Bacteroides*, derived from the mammal GIT, and the *Flavobacteria*, from aquatic environments and soil, had more than 50% of their GH-genes in clusters (SSc ≤ 5). On the contrary, most *Streptomyces*, from soil, displayed scattered GH-genes (Figure 3). Finally, *Paenibacillus* derived from multiple environments displayed intermediate GH-gene clustering. In this group, genomes from the GIT and biological fluids (i.e., milk) displayed more clustered GH-genes than genomes derived from soil and sediment (Figure 3). Finally, for each of these 128 genomes, I generated some random genomes (*n* = 5,000 iterations) with the same number of GH-genes and overall genome size. Regardless of the microbe's affiliation or environmental origin, the GH-genes in the actual genomes were systematically more clustered than their randomized counterpart (Figure 3). Thus, even in the most scattered genomes (e.g., *Streptomyces*), the co-localization of the GH-genes is not random thus suggesting that, to some degree, the clustering of GH-genes is an overarching theme affecting most bacterial lineages.

### 3.5. GH-genes colocalization with transporter genes

**Figure 4:**
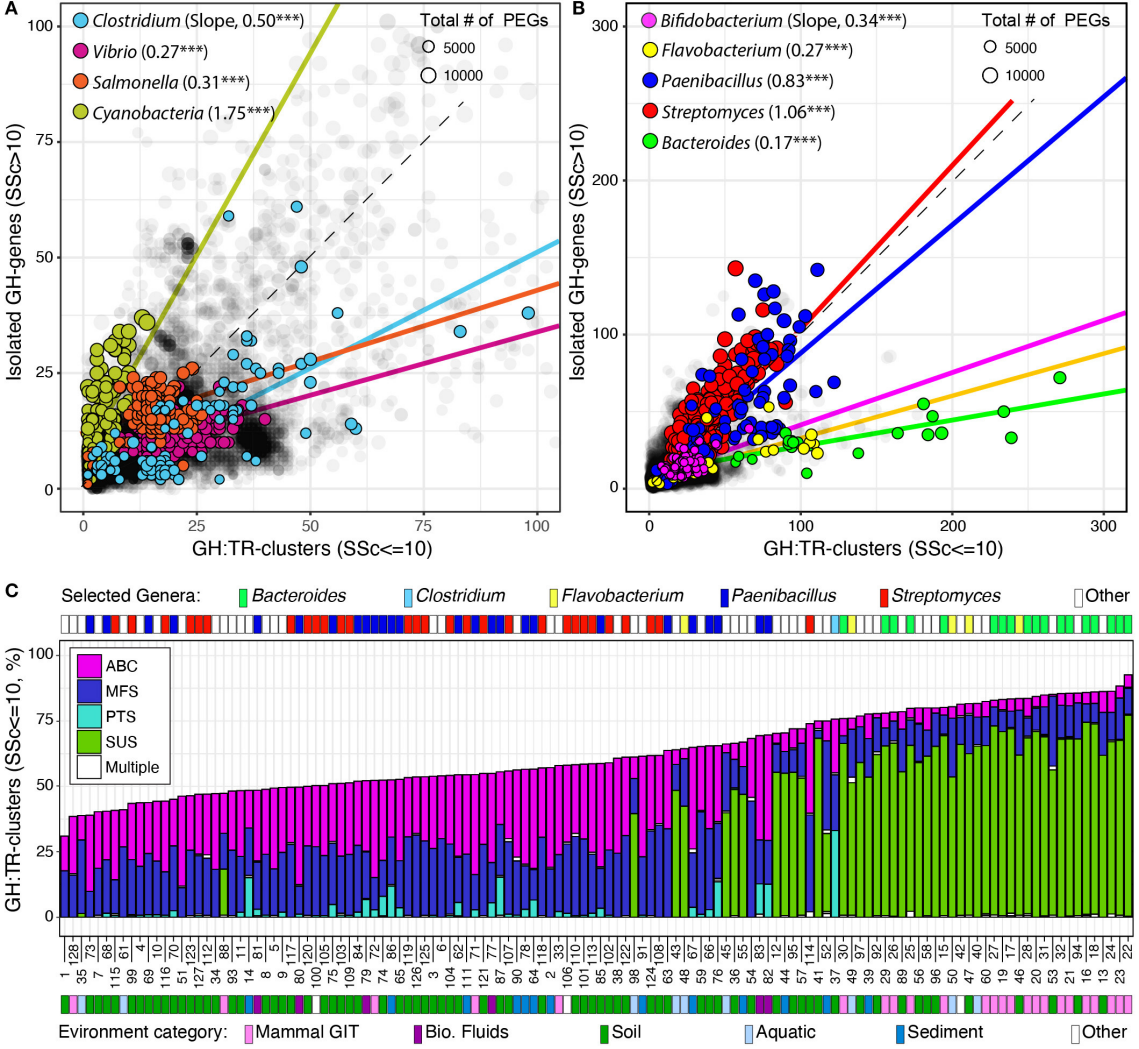
Distribution of GH-genes in GH:TR clusters or isolated in completely sequenced bacterial genomes (in gray) and in selected lineages with **(A)** reduced or **(B)** high potential for carbohydrate utilization. Frequency of the GH-genes in GH:TR clusters in 128 selected bacteria with the highest potential for carbohydrate deconstruction (i.e., > 130 identified GH-genes, **C**). For detailed information about the strains, see Supplementary Table 4.

Finally, I identified and localized genes encoding potential transporters (i.e., MFS, ABC, PTS, and SUS) and analyzed their association with previously identified GH-genes in completely sequenced genomes. I used SSc ≤ 10 to identify the clustered distribution of transporter genes with GH-genes (GH:TR-clusters, Figure 4 and Supplementary Figure 4). Globally, 57.4% of the identified GH-genes were colocalized with at least one transporter gene. However, across lineages, the number of GH:TR-clusters (SSc ≤ 10) and the type of transporter (i.e., MFS, ABC, PTS, or SUS) were highly variable.

Briefly, only 106 genomes had all their GH-genes colocalized with transporter genes. However, 97 of these genomes had < 10 GH-genes (4.7 on average). *Lactobacillus brevis* ATCC 367 (2,271 PEGs and 26 GH-genes) was the strain with the most GH-genes all being colocalized with a transporter gene (i.e., MFS and ABC only). Next, the type of transporter genes and their colocalization with GH-genes fluctuated across lineages. For example, four types of transporters were detected in the phylum Bacteroidetes, and some GH-genes were found clustered with each type (Supplementary Figure 4). However, in this phylum, most GH-genes were associated with SUS transporter gene although some were associated with ABC and MFS transporter genes. Only a few genomes, but no member of the *Bacteroidales* order had GH-genes associated with the PTS transporter gene. This uneven distribution of transporter genes and their association with GH-genes was further highlighted when considering the phylum Actinobacterium. In this group, no SUS transporter gene was identified, and the three remaining transporter types were unevenly associated with GH-genes (Supplementary Figure 4). Specifically, ~50% of the GH-genes were colocalized with ABC and MFS transporter genes, whereas < 10% were colocalized with PTS transporter genes, except in the *Coriobacteriales* order (e.g., *Coriobacterium glomerans*_PW2). This uneven distribution and association of transporter genes with GH-genes were also identified among the most abundant orders of Firmicutes and Proteobacteria (Supplementary Figure 4).

Next, the investigation of GH:TR-clusters in previously selected lineages revealed that in Cyanobacteria variation in the GH-genes content was mostly independent of the transporter genes (Figure 4A). In all the other selected lineages, but in selected Actinobacteria, variation in the GH-gene repertoire mostly mirrored fluctuation in the number of GH:TR-clusters (Figure 4). In *Streptomyces* and *Paenibacillus* (phylum Actinobacterium), the overall increase in the number of GH-genes was evenly distributed between scattered GH-genes and GH:TR-clusters (Figure 4B).

Finally, when focusing on the 128 genomes with the most identified GH-genes, < 2% of the identified GH-genes were colocalized with different types of transporter genes (Figure 4C). The genomes with the highest frequency of GH-genes in GH:TR-clusters, were Bacteroidetes (e.g., *Bacteroides* and *Flavobacteria*). In these genomes, although some GH-genes were clustered with some ABC MFS transporters, >50% of the GH:TR-clusters involved SUS transporters. In *Streptomyces* and *Paenibacilli*, the frequency of GH:TR-clusters was reduced and involved mostly ABC and MFS transporters. Few of these genomes, including some *Paenibacilli* and one *Clostridium*, had GH:TR-clusters with PTS-transporters.

## 4. Discussion

In the absence of selection, it is the frequency of the gene of interest, in this case, the GH-genes that best describes their clustering in random bacterial genomes. However, in actual genomes, variation in the frequency of GH-genes results from the interplay between the composition of the GH-gene repertoire and the overall genome size.

In most phyla, several lineages have evolved a rich and diverse GH-genes repertoire although < 1% of the analyzed PEGs are GH-genes. As predicted, this enrichment in GH-genes correlates with the fluctuation of the genome size and the frequency of clustered GH-genes. In several lineages, frequently referred to as specialized carbohydrate degraders (Stursová et al., 2012; Berlemont and Martiny, 2015; Talamantes et al., 2016; López-Mondéjar et al., 2022), a disproportionate increase in the number of GH-genes is observed. In these genomes, the frequency of observed clustered GH-genes vastly exceeds the prediction. However, this higher-than-expected frequency of clustered GH-genes is also common in genomes with a reduced number of GH-genes. This suggests that the colocalization of GH-genes in clusters is an overarching principle affecting the structure of most bacterial genomes. The physical clustering of these genes facilitates their co-expression (Junier et al., 2012), which might be beneficial to the cell when the coregulated genes support the same overall process, in this context the carbohydrate processing.

Many clustered GH-genes form codirectional segments, which could further their co-expression via transcriptional readthrough (Junier and Rivoire, 2016; Svetlitsky et al., 2020), or produce some operon. However, at least in some lineages (e.g., *Paenibacillus* see Figure 1), the uneven distribution of the GH-gene orientation can also mirror the overall chromosome organization. Indeed, having the coding strand of these GH-genes corresponding to the leading strand could prevent the occurrence of “transcription–replication conflicts” and allow for the simultaneous replication and transcription of the DNA (Wang et al., 2007; Hamperl and Cimprich, 2016). This process applies to the overall chromosome structure, not just to the GH-genes, and creates a slight bias in the number of codirectional pairs of sequential GH-genes when all the pairs of GH-genes are investigated (i.e., 57.8 vs. 50% in perfectly random genomes).

In selected lineages with a high potential for carbohydrate utilization, different trends emerged. First, most *Flavobacterium, Bacteroides*, and *Bifidobacterium* are strongly enriched in GH-genes, most of which are found in colinear clusters. Moreover, in *Bifidobacterium*, the high frequency of GH-gene mirrors both the increase in the number of GH-genes and the decrease in the total number of PEGs. In these genera, many of the identified GH-gene clusters are associated with some predicted transporter genes. The type of transporter in these GH:TR clusters is conserved within lineages, suggesting the functional coupling of the transporters with the colocalized GH-genes as described in several lineages (Terrapon et al., 2015; Ausland et al., 2021). Overall, these genomes can be considered highly clustered regarding the potential for carbohydrate processing. Next, *Paenibacillus* genomes display only some of the aforementioned genomic traits. Among others, these genomes are enriched in GH-genes, many of which are found in clusters and some of the clustered GH-genes are codirectional. However, as discussed before, this could mirror some other processes affecting the overall structure of the bacterial chromosome. In *Paenibacilli*, the frequency of GH:transporter gene cluster was intermediate. Finally, many GH-genes are also identified in *Streptomyces* although their overall frequency remains relatively low in these large genomes. In this group, most GH-genes are scattered, although some clusters are also identified. Besides the scattered GH-genes, clustered GH-genes do not display significant codirectionality. In addition, in these genomes, the frequency of GH:TR clusters was not significantly affected by the enrichment in GH-genes. Overall, these genomes can be considered scattered regarding the potential for carbohydrate processing.

In conclusion, in addition to having an enriched repertoire of GH-genes (Berlemont and Martiny, 2015; López-Mondéjar et al., 2022), bacterial carbohydrate degraders display distinct phylogenetically constrained genomic adaptations supporting carbohydrate processing. However, the distribution of these adaptations also mirrors the environmental origin of the bacterial strain. Indeed, mostly scattered genomes are associated with soil ecosystems, whereas mostly clustered genomes are derived from the mammal GIT and aquatic environments. Thus, microbes with the highest potential for carbohydrates adopted different strategies in their respective environment.

## Data availability statement

Publicly available datasets were analyzed in this study. This data can be found here: https://figshare.com/account/home#/projects/161209.

## Author contributions

This study was designed, performed, and written by RB.
