# Supplementary material for: The supragenic organization of glycoside hydrolase encoding genes reveals distinct strategies for carbohydrate utilization in bacteria
Source: Front Microbiol. 2023 Jun 2;14:1179206. doi: 10.3389/fmicb.2023.1179206 (PMC10272396; doi:10.3389/fmicb.2023.1179206)
Supplement: Supplementary file 1 [file Data_Sheet_1.docx]

Supplementary Material

The supragenic organization of glycoside hydrolase encoding genes reveals distinct strategies for carbohydrate utilization in bacteria

Renaud Berlemont^1*^

^1^Department of Biological Sciences, California State University – Long Beach, Long Beach, California, USA.

*** Correspondence:**
renaud.berlemont@csulb.edu

# Supplementary Data

Supplementary Material should be uploaded separately on submission. Please include any supplementary data, figures and/or tables.

Supplementary material is not typeset so please ensure that all information is clearly presented, the appropriate caption is included in the file and not in the manuscript, and that the style conforms to the rest of the article.

# Supplementary Figures and Tables

For more information on Supplementary Material and for details on the different file types accepted, please see [here](https://www.frontiersin.org/guidelines/author-guidelines#supplementary-material).

## Supplementary Figures


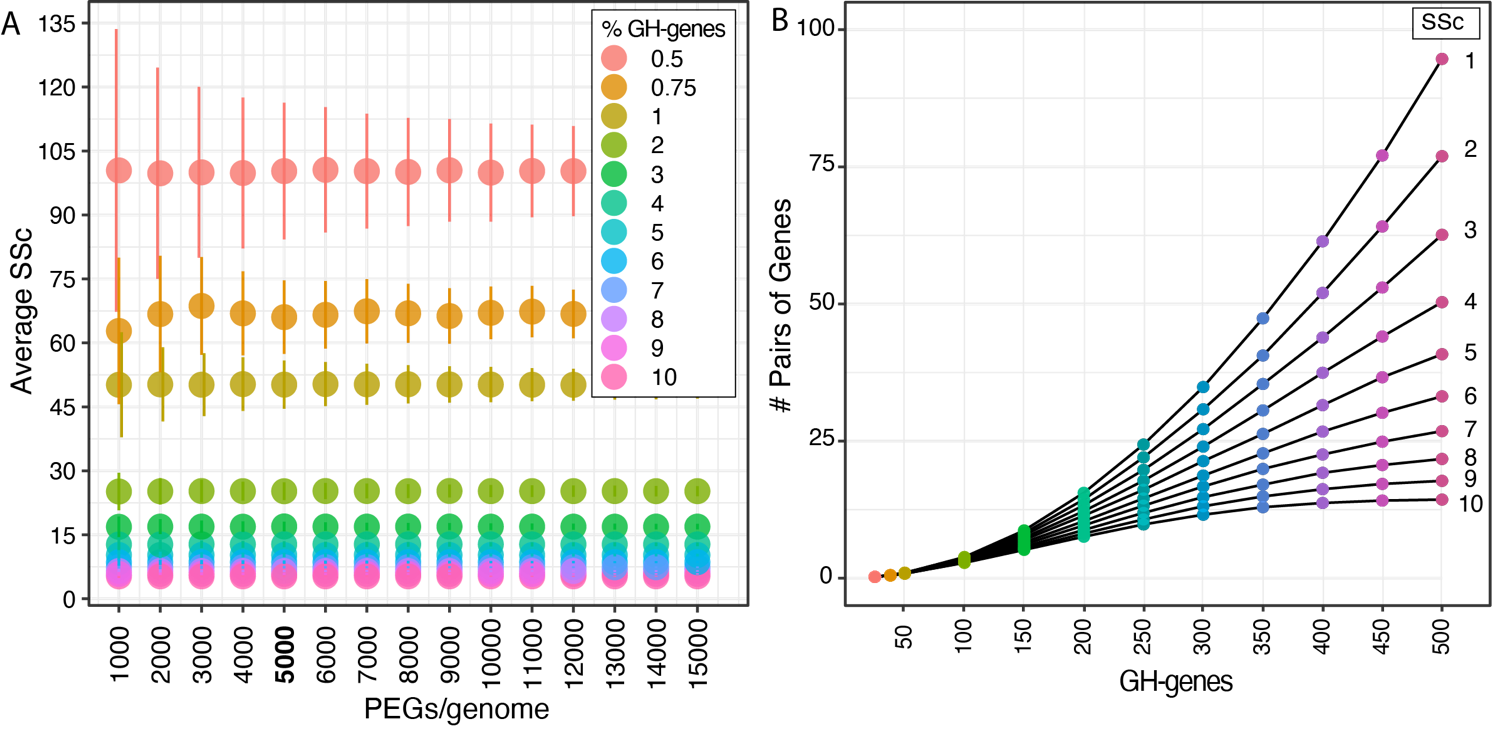


**Supplementary Figure 1.** Random distribution of genes of interest in simulated bacterial genomes. (A) Average synteny score (SSc) and standard deviation for randomized genomes with 1,000 to 15,000 PEGs and frequency of the gene of interest (i.e., GH-genes) ranging from 0.5 to 10%. (B) Number of pairs of genes of interest (i.e., GH-genes) with SSc ranging from 1 to 10 in simulated bacterial genome with 5,000 PEGs and 0.5 to 10% of gene of interest (n=5,000 iterations for each of the tested conditions).

**
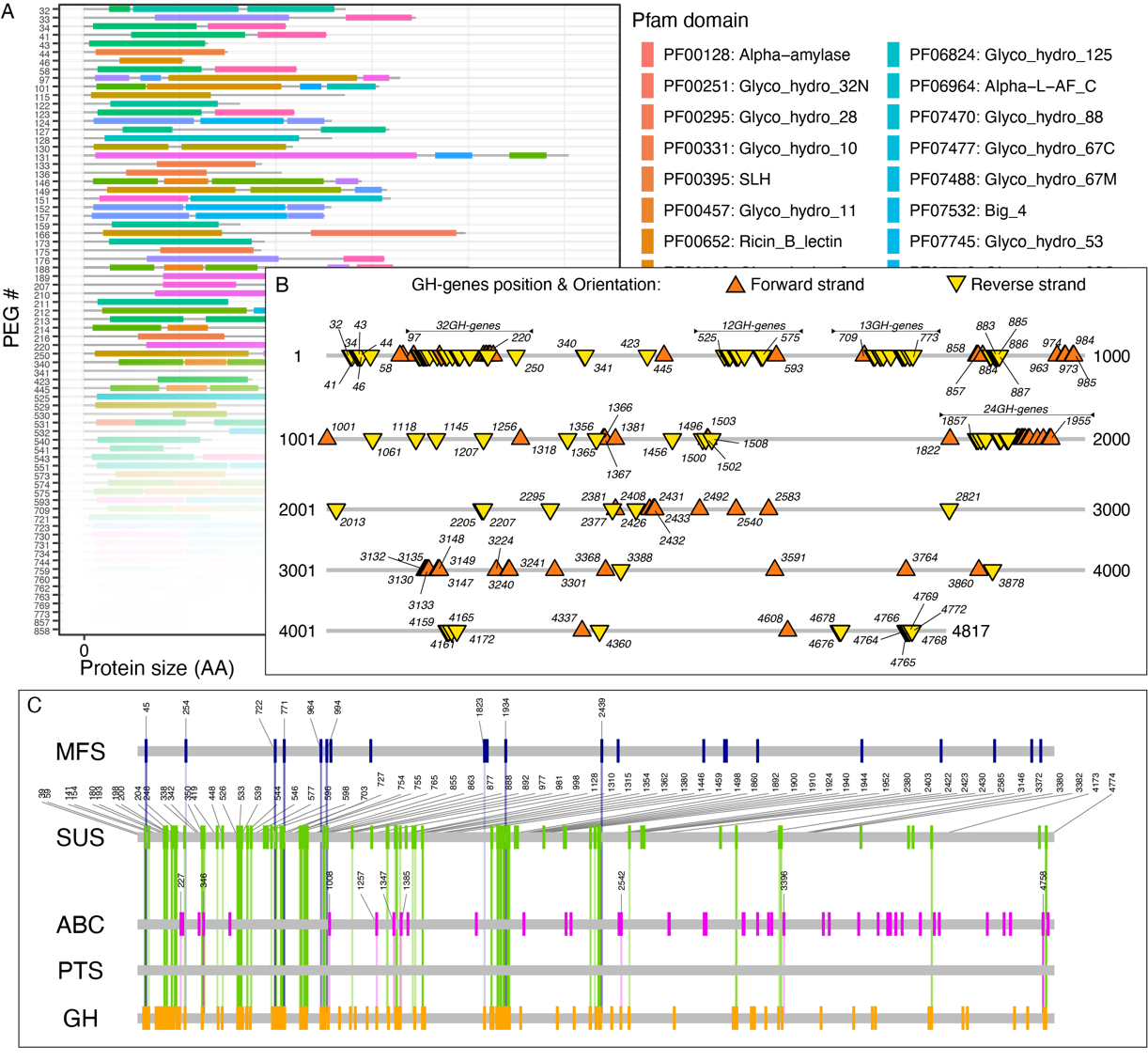
**

**Supplementary Figure 2.** *Bacteroides dorei*HS2 L 2 B 045b (4,817 predicted PEGs) analysis. (A) Domain specific identification of the GH-proteins (partial). The gene ids correspond to the PEG number as provided by the BV-BRC database. (B) Localization and orientation of the identified GH-genes on the completely sequenced genome from *B. dorei*HS2 L 2 B 045b. Numbers, when displayed, correspond to the PEG number. (C) Transporter identification and GH-gene colocalization. The vertical lines connecting the GH-genes (orange) and the transporter-genes highlight GH:TC-clusters with SSc≤10. Numbers, when displayed, correspond to the PEG number.

**
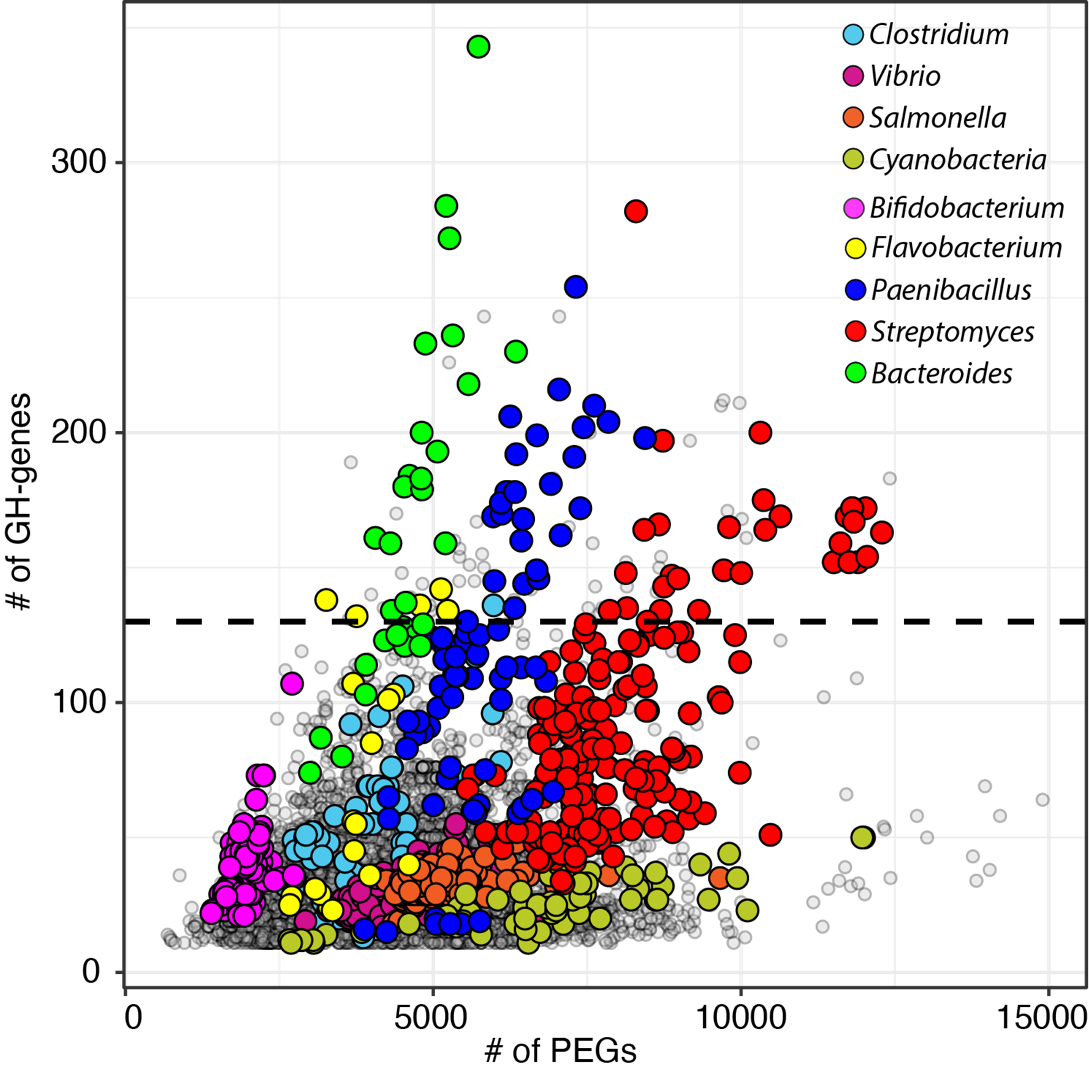
**

**Supplementary Figure 3.** Relation between the number of identified total number of protein encoding genes (PEGs, used as a proxy for the genome size) and the number of identified GH-genes in selected microbial lineages discussed in the main text. The dashed line represent the cut-off (i.e., 130 GH-genes) to select the 128 complete bacterial genome with the highest number of GH-genes.


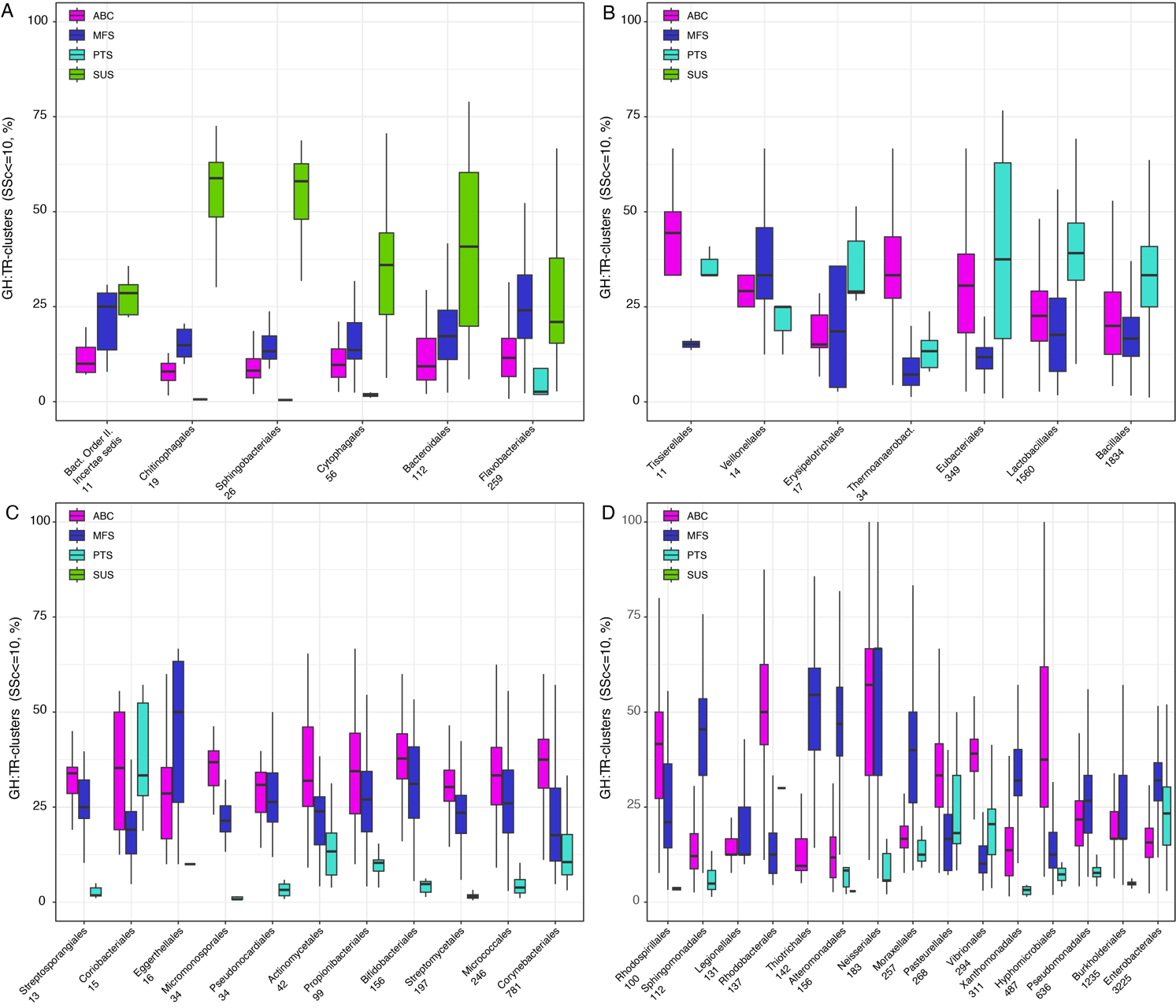


**Supplementary Figure 4.** Frequency and distribution of the GH-genes in GH:TR-clusters among the most abundant orders of Bacteroidetes (A), Firmicutes (B), Actinobacteria (C), and Proteobacteria (D). Numbers depict the numbers of completed bacterial genomes analyzed.

**Supplementary Table 1.** GH-encoding Pfam id, and description, used in this study.


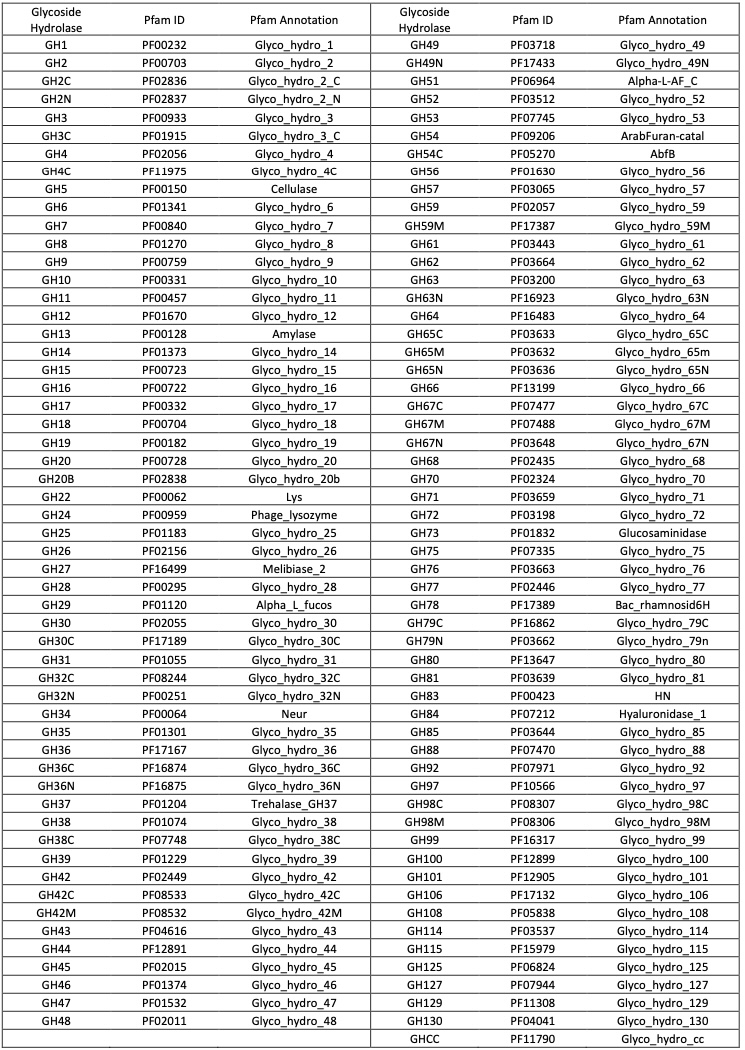


**Supplementary Table 2.** Transporter-encoding Pfam id, and description, used in this study.


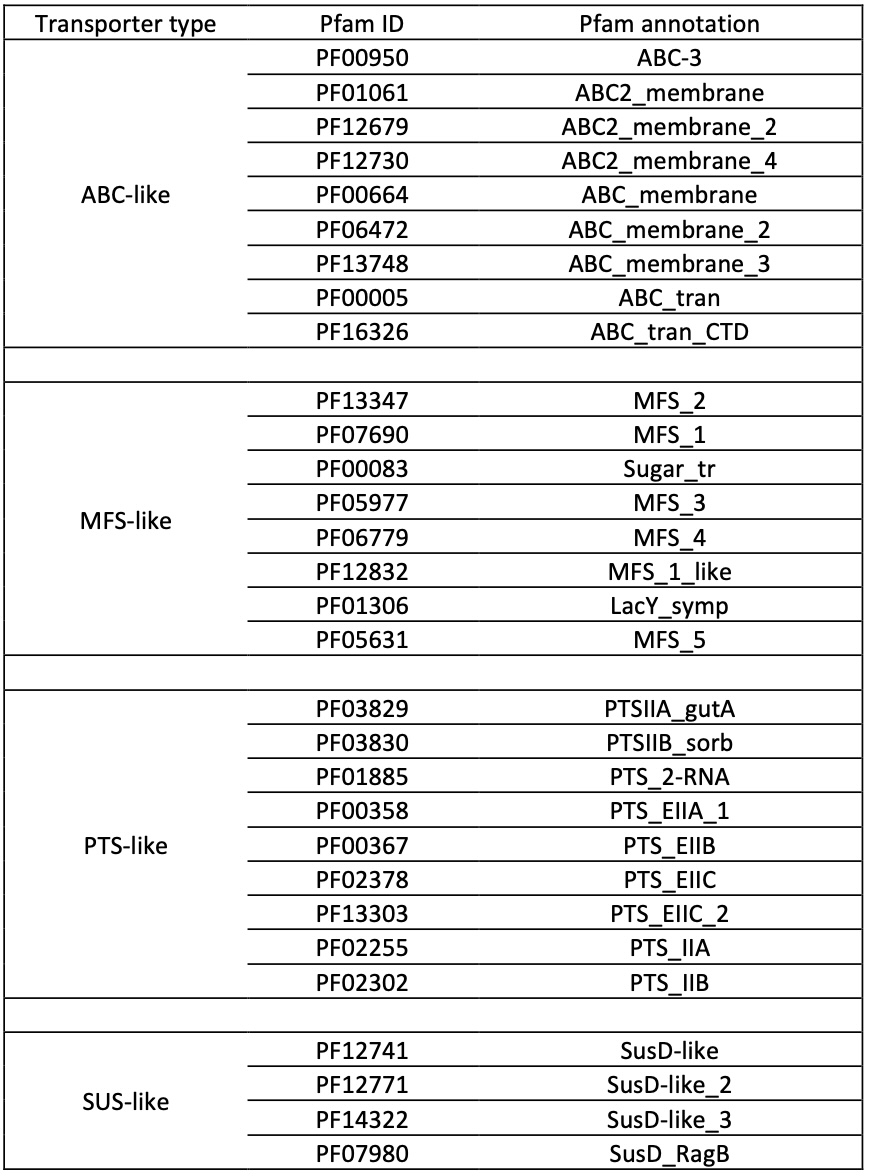


**Supplementary Table 3.** Relation between the total number of PEGs and the number of identified GH-genes in microbial genera with ≥10 completely sequenced genomes using Pearson correlation and linear regression. Percentages of the estimated variation in GH-genes clustering (SSc≤5) explained by the total number of PEGs, a proxy for the genome size, the number of GH-genes and their interaction. Estimates were derived from ANOVA model (GH-genes_SSc≤5_~PEGs×GH-genes). Significance levels P***<0.001<P**<0.01<P*<0.05< non-significant (NS).

|  | Pearson (r) | Slope | % Variance Explained by (P-value) | | | |
| --- | --- | --- | --- | --- | --- | --- |
|  |  |  | #PEGs | #GHs | #PEGs x #GHs | Res. |
| Bacteria (15,378) | 0.377*** | 0.005 | 0.0* | 1.9*** | (NS) | 98.1 |
| Actinobacteria (1,700) | 0.663*** | 0.011 | 23.6*** | 58.8*** | (NS) | 17.6 |
| *Actinomyces* (18) | 0.579* | 0.02 | 27.4*** | 66.9*** | (NS) | 5.6 |
| *Arthrobacter* (22) | 0.587** | 0.011 | 64.2*** | 22.9*** | (NS) | 12.4 |
| *Bifidobacterium* (146) | 0.447*** | 0.02 | 16.6*** | 61.1*** | 1.0* | 21.3 |
| *Clavibacter* (11) | 0.722* | 0.027 | 36.7*** | 56.9*** | (NS) | 6.1 |
| *Corynebacterium* (236) | 0.734*** | 0.004 | (NS) | (NS) | 10.0*** | 88.5 |
| *Cutibacterium* (28) | 0.872*** | 0.036 | 16.2* | (NS) | (NS) | 82.5 |
| *Gordonia* (11) | 0.914*** | 0.005 | (NS) | (NS) | (NS) | 71.9 |
| *Microbacterium* (31) | (NS) |  | 4.8 | 83.2*** | (NS) | 11.4 |
| *Micromonospora* (21) | (NS) |  | (NS) | 80.9*** | (NS) | 17.5 |
| *Mycobacterium* (401) | 0.595*** | 0.003 | (NS) | 1.3* | (NS) | 98.7 |
| *Mycobacteroides* (50) | (NS) |  | (NS) | 39.5*** | (NS) | 60.5 |
| *Mycolicibacterium* (24) | (NS) |  | 19.1* | (NS) | (NS) | 80.3 |
| *Nocardia* (15) | 0.927*** | 0.008 | (NS) | (NS) | (NS) | 68.3 |
| *Nocardioides* (14) | 0.701** | 0.005 | 31.2* | (NS) | 20.8* | 37.6 |
| *Propionibacterium* (26) | 0.833*** | 0.004 | (NS) | 80.6*** | (NS) | 16.3 |
| *Rhodococcus* (48) | 0.610*** | 0.001 | 18.0** | 16.6** | (NS) | 65.4 |
| *Streptomyces* (199) | 0.622*** | 0.018 | 19.3*** | 40.7*** | (NS) | 39.9 |
| Bacteroidetes (489) | 0.649*** | 0.029 | 27.8*** | 67.7*** | (NS) | 4.4 |
| *Bacteroides* (30) | 0.792*** | 0.071 | 57.5*** | 41.9*** | (NS) | 0.6 |
| *Capnocytophaga* (21) | (NS) |  | (NS) | 82.5*** | (NS) | 17.5 |
| *Chryseobacterium* (45) | 0.574*** | 0.008 | 15.6*** | 77.6*** | 1.2** | 5.7 |
| *Elizabethkingia* (25) | 0.870*** | 0.007 | 20.6** | (NS) | 47.6*** | 31.2 |
| *Flavobacterium* (40) | 0.845*** | 0.049 | 65.6*** | 33.9*** | 0.1** | 0.4 |
| *Hymenobacter* (11) | 0.696* | 0.037 | 43.9*** | 54.7*** | (NS) | 1.4 |
| *Porphyromonas* (23) | 0.558** | 0.012 | 29.5*** | 65.1*** | 1.6** | 3.9 |
| *Prevotella* (22) | (NS) |  | 1.4** | 96.2*** | (NS) | 1.9 |
| *Riemerella* (12) | 0.601* | 0.01 | 51.0*** | 46.8*** | 0.0* | 2.2 |
| Chlamydiae  (29) | 0.756*** | 0.002 | 24.6 | 49.3*** | 0.0* | 26.1 |
| *Chlamydia* (24) | (NS) |  | NA | NA | NA | NA |
| Cyanobacteria (169) | 0.871*** | 0.004 | 24.4*** | 21.7*** | (NS) | 53.8 |
| *Nostoc* (15) | 0.703** | 0.003 | (NS) | (NS) | (NS) | 89.1 |
| *Prochlorococcus* (16) | 0.853*** | 0.002 | NA | NA | NA | NA |
| *Synechococcus* (37) | (NS) |  | (NS) | 33.0*** | (NS) | 61.9 |
| Deinococcus*-Thermus*(35) | 0.730*** | 0.011 | 10.8* | 17.1*** | (NS) | 69.3 |
| *Deinococcus* (18) | 0.569* | 0.012 | 16.5** | 70.7*** | (NS) | 12.4 |
| Firmicutes (3,884) | 0.381*** | 0.007 | 5.8*** | 74.7*** | 0.0** | 19.4 |
| *Bacillus* (699) | -0.325*** | -0.004 | 6.4*** | 66.1*** | 1.2*** | 26.3 |
| *Carnobacterium* (13) | 0.929*** | 0.023 | 83.3*** | 13.9*** | 1.9** | 1.0 |
| *Clostridioides* (76) | (NS) |  | (NS) | 63.7*** | 11.8 | 24.5 |
| *Clostridium* (127) | 0.459*** | 0.015 | 17.2*** | 42.2*** | (NS) | 40.5 |
| *Enterococcus* (220) | 0.604*** | 0.02 | 18.9*** | 48.0*** | (NS) | 33.0 |
| *Geobacillus* (27) | (NS) |  | 2.9* | 83.7*** | (NS) | 13.4 |
| *Lactobacillus* (423) | 0.775*** | 0.018 | 47.9*** | 34.6*** | 0.4** | 17.0 |
| *Lactococcus* (53) | 0.411** | 0.01 | (NS) | 68.4*** | 5.8** | 24.3 |
| *Leuconostoc* (39) | 0.726*** | 0.026 | 28.0*** | 43.6*** | (NS) | 25.9 |
| *Listeria* (221) | 0.306*** | 0.006 | (NS) | 5.9*** | (NS) | 93.3 |
| *Lysinibacillus* (16) | (NS) |  | NA | NA | NA | NA |
| *Paenibacillus* (79) | 0.683*** | 0.043 | 39.5*** | 56.0*** | 0.3* | 4.2 |
| *Pediococcus* (36) | 0.678*** | 0.022 | 49.7*** | 23.5*** | 5.4** | 21.4 |
| *Staphylococcus* (709) | 0.107** | 0.001 | 1.1** | 21.2*** | (NS) | 77.4 |
| *Streptococcus* (730) | 0.364*** | 0.014 | 3.7*** | 31.2*** | 0.7** | 64.4 |
| *Weissella* (24) | 0.841*** | 0.016 | 32.7*** | 47.1*** | (NS) | 17.9 |
| Fusobacteria (45) | 0.419** | 0.008 | 15.1*** | 70.3*** | (NS) | 13.3 |
| *Fusobacterium* (33) | 0.466** | 0.004 | 2.6** | 86.4*** | (NS) | 11.0 |
| Proteobacteria (8,243) | 0.433*** | 0.006 | (NS) | 1.7*** | (NS) | 98.2 |
| Alphaproteobacteria  (985) | 0.502*** | 0.004 | (NS) | 1.1** | 0.9** | 97.8 |
| *Acetobacter* (21) | 0.794*** | 0.005 | 70.8*** | 14.2*** | 14.2*** | 0.8 |
| *Agrobacterium* (25) | 0.870*** | 0.006 | 29.1** | 12.5* | (NS) | 55.2 |
| *Altererythrobacter* (11) | (NS) |  | (NS) | 89.4*** | (NS) | 10.2 |
| *Azospirillum* (15) | 0.883*** | 0.005 | 19.9** | 69.9*** | (NS) | 9.7 |
| *Bartonella* (26) | 0.436* | 0.002 | (NS) | (NS) | (NS) | 100.0 |
| *Bradyrhizobium* (31) | 0.445* | 0.001 | 20.3** | 18.8** | (NS) | 58.0 |
| *Brevundimonas* (13) | (NS) |  | 5.2** | 90.4*** | (NS) | 4.1 |
| *Brucella* (162) | (NS) |  | 3.5*** | 68.8*** | 7.5*** | 20.1 |
| *Caulobacter* (12) | 0.706* | 0.019 | 35.1*** | 58.9*** | (NS) | 3.8 |
| *Erythrobacter* (15) | (NS) |  | 13.3* | 59.4*** | (NS) | 26.6 |
| *Mesorhizobium* (29) | 0.781*** | 0.003 | (NS) | (NS) | (NS) | 88.0 |
| *Methylobacterium* (18) | 0.833*** | 0.004 | 34.7** | (NS) | (NS) | 63.8 |
| *Paracoccus* (16) | (NS) |  | (NS) | 77.2*** | (NS) | 22.8 |
| *Phaeobacter* (34) | (NS) |  | (NS) | 33.1** | (NS) | 66.9 |
| *Rhizobium* (49) | 0.700*** | 0.007 | 19.5*** | 31.8*** | (NS) | 48.8 |
| *Rhodobacter* (19) | 0.483* | 0.004 | (NS) | (NS) | (NS) | 78.6 |
| *Rickettsia* (56) | 0.282* | 0.001 | NA | NA | NA | NA |
| *Sinorhizobium* (27) | 0.913*** | 0.004 | (NS) | 25.5** | (NS) | 70.5 |
| *Sphingobium* (17) | (NS) |  | 4.4* | 86.7*** | (NS) | 8.8 |
| *Sphingomonas* (24) | (NS) |  | 1.9** | 95.6*** | (NS) | 2.6 |
| *Sphingopyxis* (15) | (NS) |  | (NS) | 97.3*** | (NS) | 1.9 |
| Betaproteobacteria (1,474) | 0.621*** | 0.004 | 10.3*** | 48.1*** | 1.8*** | 39.8 |
| *Achromobacter* (29) | (NS) |  | 18.6** | 26.0*** | 14.9** | 40.5 |
| *Acidovorax* (30) | (NS) |  | (NS) | 30.4** | (NS) | 65.8 |
| *Bordetella* (624) | 0.652*** | 0.003 | 0.9** | 37.9*** | 7.5*** | 53.8 |
| *Burkholderia* (280) | 0.494*** | 0.003 | 3.6*** | 14.6*** | 5.7*** | 76.2 |
| *Cupriavidus* (22) | (NS) |  | (NS) | 26.2* | (NS) | 73.8 |
| *Massilia* (12) | 0.695* | 0.047 | 32.2*** | 64.4*** | (NS) | 2.3 |
| *Neisseria* (158) | 0.190* | 0.001 | 14.2*** | 16.7*** | 9.7*** | 59.4 |
| *Pandoraea* (21) | (NS) |  | 23.8** | 27.8** | (NS) | 44.5 |
| *Paraburkholderia* (24) | (NS) |  | (NS) | 38.9** | (NS) | 56.3 |
| *Ralstonia* (59) | 0.294* | 0.002 | 9.7* | 7.5* | (NS) | 82.8 |
| Gammaproteobacteria (5,765) | 0.449*** | 0.006 | 0.1** | 1.8*** | (NS) | 98.0 |
| *Acinetobacter* (258) | 0.625*** | 0.004 | 6.3** | (NS) | (NS) | 93.7 |
| *Actinobacillus* (14) | 0.589* | 0.012 | (NS) | 36.1* | (NS) | 51.8 |
| *Aeromonas* (52) | (NS) |  | 4.6* | 52.5*** | (NS) | 42.7 |
| *Aggregatibacter* (15) | (NS) |  | (NS) | 69.4*** | (NS) | 27.4 |
| *Alteromonas* (38) | (NS) |  | 12.9*** | 72.9*** | 3.2** | 11.0 |
| *Citrobacter* (77) | 0.404*** | 0.003 | (NS) | 38.1*** | (NS) | 61.3 |
| *Coxiella* (20) | 0.500* | 0.002 | (NS) | (NS) | (NS) | 91.6 |
| *Cronobacter* (16) | 0.968*** | 0.012 | 93.6*** | (NS) | (NS) | 4.3 |
| *Dickeya* (19) | 0.551* | 0.01 | (NS) | (NS) | (NS) | 85.4 |
| *Enterobacter* (144) | (NS) |  | 3.3* | 5.4** | (NS) | 89.6 |
| *Escherichia* (1,063) | 0.609*** | 0.005 | (NS) | (NS) | (NS) | 99.4 |
| *Francisella* (83) | (NS) |  | 41.9*** | (NS) | (NS) | 56.9 |
| *Haemophilus* (82) | 0.493*** | 0.005 | 5.8** | 43.7*** | (NS) | 48.2 |
| *Halomonas* (24) | 0.491* | 0.001 | 30.3*** | 31.3*** | 12.1** | 26.3 |
| *Histophilus* (16) | (NS) |  | 6.3** | 75.5*** | 6.3* | 12.0 |
| *Klebsiella* (521) | 0.390*** | 0.003 | 21.5*** | 15.6*** | 2.8*** | 60.1 |
| *Leclercia* (12) | 0.852*** | 0.003 | 61.2** | 1.8 | (NS) | 27.0 |
| *Legionella* (110) | 0.884*** | 0.004 | NA | NA | NA | NA |
| *Mannheimia* (71) | 0.499*** | 0.005 | (NS) | 38.0*** | 5.4* | 54.5 |
| *Marinobacter* (18) | (NS) |  | (NS) | 51.1** | (NS) | 38.7 |
| *Morganella* (12) | (NS) |  | NA | NA | NA | NA |
| *Pantoea* (26) | 0.583 | 0.007 | (NS) | (NS) | (NS) | 78.1 |
| *Pasteurella* (52) | 0.519*** | 0.005 | (NS) | (NS) | (NS) | 87.8 |
| *Pectobacterium* (29) | (NS) |  | (NS) | 15.3* | (NS) | 74.9 |
| *Piscirickettsia* (45) | 0.347* | 0.001 | NA | NA | NA | NA |
| *Proteus* (39) | (NS) |  | (NS) | (NS) | (NS) | 100.0 |
| *Pseudoalteromonas* (34) | 0.645*** | 0.009 | (NS) | 28.2** | (NS) | 66.9 |
| *Pseudomonas* (624) | (NS) |  | 16.8*** | 42.1*** | 2.1*** | 39.0 |
| *Raoultella* (17) | 0.701** | 0.005 | 53.3*** | 30.6*** | 9.6** | 6.5 |
| *Salmonella* (888) | 0.650*** | 0.006 | (NS) | (NS) | (NS) | 99.8 |
| *Serratia* (97) | 0.310** | 0.005 | 13.5*** | 35.5*** | 4.1** | 46.8 |
| *Shewanella* (47) | (NS) |  | 3.4** | 76.6*** | (NS) | 20.0 |
| *Shigella* (112) | (NS) |  | 20.5*** | 8.0** | (NS) | 71.0 |
| *Stenotrophomonas* (65) | -0.509*** | -0.004 | (NS) | (NS) | (NS) | 89.6 |
| *Vibrio* (291) | 0.671*** | 0.007 | (NS) | 4.3*** | (NS) | 95.4 |
| *Xanthomonas* (208) | (NS) |  | (NS) | 3.6** | (NS) | 94.0 |
| *Xylella* (18) | (NS) |  | (NS) | 26.2* | (NS) | 60.7 |
| *Yersinia* (101) | 0.346*** | 0.003 | (NS) | (NS) | (NS) | 95.2 |
| *delta/*epsilon *subdivisions*(175) | 0.797*** | 0.006 | 45.2*** | 38.8*** | 1.0** | 15.0 |
| *Arcobacter* (16) | (NS) |  | 0.0*** | 96.9*** | 0.0*** | 3.1 |
| *Campylobacter* (43) | (NS) |  | (NS) | (NS) | (NS) | 100.0 |
| *Desulfovibrio* (15) | (NS) |  | (NS) | 29.1* | (NS) | 61.3 |
| *Geobacter* (15) | (NS) |  | (NS) | 69.1*** | (NS) | 24.8 |
| Spirochaetes  (160) | 0.289*** | 0.002 | 1.9*** | 83.4*** | 1.9*** | 12.8 |
| *Borrelia* (29) | (NS) |  | (NS) | (NS) | (NS) | 100.0 |
| *Leptospira* (49) | -0.298* | -0.001 | 14.1** | (NS) | (NS) | 82.3 |
| *Treponema* (40) | 0.842*** | 0.012 | 40.5*** | 51.7*** | 1.3* | 6.5 |
| Tenericutes (137) | 0.293* | 0.005 | (NS) | 12.8*** | 17.6*** | 67.9 |
| *Mesoplasma* (17) | (NS) |  | 22.5** | 53.5*** | (NS) | 20.9 |
| *Mycoplasma* (82) | (NS) |  | (NS) | 74.2*** | 1.6* | 23.4 |
| *Spiroplasma* (24) | (NS) |  | (NS) | 65.8*** | 11.1** | 21.1 |
| Thermotogae (32) | (NS) |  | (NS) | 19.0*** | (NS) | 76.4 |
| *Thermotoga* (13) | -0.715** | -0.054 | (NS) | (NS) | (NS) | 81.5 |
| Verrucomicrobia (41) | 0.842*** | 0.038 | 67.5*** | 23.2*** | (NS) | 9.0 |
| *Akkermansia* (27) | (NS) |  | (NS) | (NS) | (NS) | 88.2 |
|  | | | | | | |
| Archaea (262) | 0.204*** | 0.002 | 4.7*** | 27.3*** | (NS) | 67.9 |
| Crenarchaeota (82) | 0.404*** | 0.004 | 10.3*** | 60.8*** | 2.8** | 26.0 |
| *Sulfolobus* (25) | 0.887*** | 0.012 | 32.8*** | 49.6*** | 5.9** | 11.6 |
| Euryarchaeota (169) | 0.193* | 0.002 | 3.7** | 27.2*** | (NS) | 69.2 |
| *Methanosarcina* (28) | (NS) |  | 11.3** | 39.6*** | 34.0*** | 15.0 |
| *Thermococcus* (29) | (NS) |  | 9.0* | 48.6*** | (NS) | 41.5 |

**Supplementary Table 4.** Identification of the 128 completely sequenced bacterial genomes with the highest number of identified GH-genes (i.e., >130GH-genes). For each genome, information about the environmental origin of the bacterium was manually retrieved from the BV-RBC database (https://www.bv-brc.org), the Leibniz Institute (DSMZ, https://www.dsmz.de), the Joint Genome Institute (JGI, https://jgi.doe.gov), or individual publications (DOI provided).

| # | Strain (Genome ID) | Origin-environmental category  (information provided by) |
| --- | --- | --- |
| 1 | *Acidobacteriaceae bacterium* SBC82 (GID: 2211140.3) | soil (BV-BRC) |
| 2 | *Actinoplanes derwentensis* DSM 43941 (GID: 113562.5) | aquatic sediment (DSMZ) |
| 3 | *Actinoplanes friuliensis* DSM 7358 (GID: 1246995.3) | soil (BV-BRC) |
| 4 | *Actinoplanes missouriensis* 431 (GID: 512565.3) | soil (DOI: [10.4056/sigs.3196539](https://doi.org/10.4056/sigs.3196539)) |
| 5 | *Actinoplanes* sp*.* N902-109 (GID: 649831.3) | soil DOI: [10.7164/antibiotics.48.657](https://doi.org/10.7164/antibiotics.48.657) |
| 6 | *Actinoplanes* sp*.* OR16 (GID: 946334.3) | soil (BV-BRC) |
| 7 | *Actinoplanes* sp*.* SE50 (GID: 2033844.3) | soil (BV-BRC) |
| 8 | *Actinoplanes teichomyceticus* ATCC 31121 (GID: 457423.5) | soil (BV-BRC) |
| 9 | *Amycolatopsis mediterranei* RB (GID: 1221524.3) | soil (DOI: [10.1007/s12088-016-0590-8](https://doi.org/10.1007/s12088-016-0590-8)) |
| 10 | *Amycolatopsis mediterranei* S699 (GID: 713604.12) | soil (BV-BRC) |
| 11 | *Amycolatopsis mediterranei* U32 (GID: 749927.5) | soil (DOI: 10.1007/BF02089930) |
| 12 | *Arachidicoccus ginsenosidivorans* Gsoil 809 (GID: 496057.3) | soil (BV-BRC) |
| 13 | *Arachidicoccus* sp*.* KIS59-12 (GID: 2341117.3) | soil (BV-BRC) |
| 14 | *Bacillus foraminis* Bac44 (GID: 279826.4) | aquatic sediment (BV-BRC) |
| 15 | *Bacteroides cellulosilyticus* WH2 (GID: 246787.4) | GIT (BV-BRC) |
| 16 | *Bacteroides dorei* CL03T12C01 (GID: 997877.5) | GIT (BV-BRC) |
| 17 | *Bacteroides dorei* HS1_L_1_B_010 (GID: 357276.36) | GIT (BV-BRC) |
| 18 | *Bacteroides dorei* HS1_L_3_B_079 (GID: 357276.37) | GIT (BV-BRC) |
| 19 | *Bacteroides dorei* HS2_L_2_B_045b (GID: 357276.38) | GIT (BV-BRC) |
| 20 | *Bacteroides dorei* MGYG-HGUT-02478 (GID: 357276.951) | GIT (BV-BRC) |
| 21 | *Bacteroides ovatus* 3725 D1 iv (GID: 28116.1147) | GIT (BV-BRC) |
| 22 | *Bacteroides ovatus* FDAARGOS_733Not applicable (GID: 28116.1185) | GIT (BV-BRC) |
| 23 | *Bacteroides ovatus* V975 (GID: 1379690.3) | GIT (BV-BRC) |
| 24 | *Bacteroides* sp*.* A1C1 (GID: 2528203.4) | GIT (BV-BRC) |
| 25 | *Bacteroides* sp*.* I48 (GID: 1796613.4) | GIT (BV-BRC) |
| 26 | *Bacteroides thetaiotaomicron* 7330 (GID: 818.23) | GIT (BV-BRC) |
| 27 | *Bacteroides thetaiotaomicron* VPI-5482 (GID: 226186.12) | GIT (BV-BRC) |
| 28 | *Bacteroides vulgatus* ATCC 8482 (GID: 435590.9) | GIT (BV-BRC) |
| 29 | *Bacteroides vulgatus* mpk (GID: 821.4) | GIT (BV-BRC) |
| 30 | *Bacteroides vulgatus* VIC01 (GID: 821.3445) | GIT (BV-BRC) |
| 31 | *Bacteroides xylanisolvens* H207 (GID: 371601.430) | GIT (BV-BRC) |
| 32 | *Bacteroides xylanisolvens* XB1A (GID: 657309.4) | GIT (BV-BRC) |
| 33 | *Blautia producta* PMF1 (GID: 33035.72) | GIT (BV-BRC) |
| 34 | *Catenulispora acidiphila* DSM 44928 (GID: 479433.5) | soil (BV-BRC) |
| 35 | *Cellvibrio* sp*.* PSBB006PSBB006 (GID: 1987723.3) | aquatic (BV-BRC) |
| 36 | *Chitinophaga pinensis* DSM 2588 (GID: 485918.6) | soil (BV-BRC) |
| 37 | *Clostridium* saccharoperbutylacetonicum N1-4 (HMT) (GID: 931276.5) | soil (DOI: [10.1099/00207713-45-4-693](https://doi.org/10.1099/00207713-45-4-693)) |
| 38 | *Cohnella* sp*.* 18JY8-7 (GID: 2480923.3) | soil (BV-BRC) |
| 39 | *Draconibacterium orientale* FH5 (GID: 1168034.5) | aquatic sediment (BV-BRC) |
| 40 | *Echinicola rosea* JL3085 (GID: 1807691.3) | aquatic (BV-BRC) |
| 41 | *Echinicola sp.* LN3S3 (GID: 2591634.3) | soil (BV-BRC) |
| 42 | *Echinicola strongylocentroti* MEBiC08714 (GID: 1795355.3) | sea urchin (DOI: [10.1099/ijsem.0.001691](https://doi.org/10.1099/ijsem.0.001691)) |
| 43 | *Filimonas lacunae* NBRC 104114 (GID: 477680.5) | aquatic (BV-BRC) |
| 44 | *Flammeovirga* sp*.* MY04 (GID: 1191459.5) | aquatic sediment (BV-BRC) |
| 45 | *Flavobacteriaceae bacterium* 10Alg115 (GID: 2584122.3) | aquatic (BV-BRC) |
| 46 | *Flavobacterium johnsoniae* UW101 (GID: 376686.1) | soil (BV-BRC) |
| 47 | *Flavobacterium nitrogenifigens* KACC 18538 (GID: 1617283.4) | soil (BV-BRC) |
| 48 | *Flavobacterium* sp*.* EM1308 (GID: 1492737.5) | aquatic (BV-BRC) |
| 49 | *Flavobacterium* sp*.* HYN0056 (GID: 2183896.3) | aquatic (DOI: [10.1099/ijsem.0.003067](https://doi.org/10.1099/ijsem.0.003067)) |
| 50 | *Flavobacterium* sp*.* PK15 (GID: 1306519.4) | aquatic (BV-BRC) |
| 51 | *Lentzea* sp*.* DHS C013 (GID: 1586287.3) | soil (BV-BRC) |
| 52 | *Marinifilaceae bacterium* SPP2 (GID: 1717717.3) | aquatic sediment (BV-BRC) |
| 53 | *Mariniflexile* sp*.* TRM1-10 (GID: 2027857.3) | soil (BV-BRC) |
| 54 | *Massilia lutea*DSM 17473 (GID: 321985.3) | soil (BV-BRC) |
| 55 | *Mucilaginibacter ginsenosidivorax* KHI28 (GID: 862126.3) | aquatic sediment (BV-BRC) |
| 56 | *Mucilaginibacter mallensis* MP1X4 (GID: 652787.3) | soil (DSMZ) |
| 57 | *Niabella ginsenosidivorans* BS26 (GID: 1176587.6) | soil (BV-BRC) |
| 58 | *Niastella koreensis* GR20-10 (GID: 700598.3) | soil (BV-BRC) |
| 59 | *Nonomuraea* sp*.* ATCC 55076ATCC 55076 (GID: 1909395.3) | soil (BV-BRC) |
| 60 | *Olivibacter* sp*.* LS-1 (GID: 2592345.3) | GIT (BV-BRC) |
| 61 | *Opitutus terrae* PB90-1 (GID: 452637.7) | aquatic (BV-BRC) |
| 62 | *Paenibacillus borealis* DSM 13188 (GID: 160799.4) | soil (BV-BRC) |
| 63 | *Paenibacillus cellulositrophicus* KACC 16577 (GID: 562959.3) | soil (BV-BRC) |
| 64 | *Paenibacillus donghaensis* KCTC 13049 (GID: 414771.3) | aquatic sediment (BV-BRC) |
| 65 | *Paenibacillus graminis* DSM 15220 (GID: 189425.5) | soil (BV-BRC) |
| 66 | *Paenibacillus guangzhouensis* KCTC 33171 (GID: 1473112.3) | soil (BV-BRC) |
| 67 | *Paenibacillus ihbetae* IHBB 9852 (GID: 1870820.3) | aquatic sediment (BV-BRC) |
| 68 | *Paenibacillus mucilaginosus* 3016 (GID: 1116391.3) | soil (BV-BRC) |
| 69 | *Paenibacillus mucilaginosus* K02 (GID: 997761.3) | soil (DOI: [10.1016/j.micpath.2016.01.016](https://doi.org/10.1016/j.micpath.2016.01.016)) |
| 70 | *Paenibacillus mucilaginosus* KNP414 (GID: 1036673.3) | soil (DOI: [10.1128/genomeA.00881-13](https://doi.org/10.1128/genomea.00881-13)) |
| 71 | *Paenibacillus odorifer* CBA7130 (GID: 189426.45) | GIT (BV-BRC) |
| 72 | *Paenibacillus odorifer* MGYG-HGUT-02414 (GID: 189426.48) | GIT (BV-BRC) |
| 73 | *Paenibacillus psychroresistens* ML311-T8 (GID: 1778678.3) | soil (BV-BRC) |
| 74 | *Paenibacillus riograndensis* SBR5 (GID: 1073571.4) | soil (BV-BRC) |
| 75 | *Paenibacillus* sp*.* BIHB4019 (GID: 1870819.3) | soil (BV-BRC) |
| 76 | *Paenibacillus* sp*.* DCT19rhisosphere (GID: 2211212.3) | soil (BV-BRC) |
| 77 | *Paenibacillus* sp*.* FSL H7-0357 (GID: 1536774.3) | biological fluid (milk) (BV-BRC) |
| 78 | *Paenibacillus* sp*.* FSL H7-0737 (GID: 1536775.3) | biological fluid (milk) (BV-BRC) |
| 79 | *Paenibacillus* sp*.* FSL P4-0081 (GID: 1536769.3) | biological fluid (milk) (BV-BRC) |
| 80 | *Paenibacillus sp.* FSL R5-0345 (GID: 1536770.3) | biological fluid (milk) (BV-BRC) |
| 81 | *Paenibacillus* sp*.* FSL R5-0912 (GID: 1536771.3) | biological fluid (milk) (BV-BRC) |
| 82 | *Paenibacillus* sp*.* FSL R7-0273 (GID: 1536772.3) | biological fluid (milk) (BV-BRC) |
| 83 | *Paenibacillus* sp*.* FSL R7-0331 (GID: 1536773.3) | biological fluid (milk) (BV-BRC) |
| 84 | *Paenibacillus* sp*.* JDR-2 (GID: 324057.4) | soil (BV-BRC) |
| 85 | *Paenibacillus* sp*.* Y412MC10 (GID: 481743.5) | soil (BV-BRC) |
| 86 | *Paenibacillus xylanexedens* PAMC 22703 (GID: 528191.3) | aquatic sediment (BV-BRC) |
| 87 | *Paenibacillus xylanilyticus* W4 (GID: 248903.4) | soil (BV-BRC) |
| 88 | *Paraprevotella xylaniphila* 82A6 (GID: 762982.68) | GIT (JGI) |
| 89 | *Pedobacter suwonensis* Fj_001 (GID: 332999.9) | Soil (BV-BRC) |
| 90 | *Plantactinospora* sp*.* BB1 (GID: 2071627.3) | aquatic sediment (BV-BRC) |
| 91 | *Plantactinospora* sp*.* BC1 (GID: 2108470.3) | aquatic sediment (DOI: [10.1128/genomeA.00436-18](https://doi.org/10.1128/genomea.00436-18)) |
| 92 | P*seudoflavitalea* sp. 5GH32-13 (GID: 2315862.3) | soil (BV-BRC) |
| 93 | *Saccharothrix syringae*NRRL B-16468 (GID: 103733.7) | soil (BV-BRC) |
| 94 | *Sphingobacteriaceae bacterium* GW460-11-11-14-LB5 (GID: 1986952.3) | soil (BV-BRC) |
| 95 | *Sphingobacterium* sp. 21 (GID: 743722.3) | soil (JGI) |
| 96 | *Sphingobacterium* sp. B29B29 (GID: 1933220.4) | soil (BV-BRC) |
| 97 | *Sphingobacterium* sp. G1-14G1-14 (GID: 2003121.3) | soil (BV-BRC) |
| 98 | *Spirosoma* sp. KCTC 42546 (GID: 2520506.3) | aquatic (BV-BRC) |
| 99 | *Streptomyces bingchenggensis* BCW-1 (GID: 749414.3) | soil (BV-BRC) |
| 100 | *Streptomyces brunneus* CR22 (GID: 2495589.3) | NA |
| 101 | *Streptomyces chartreusis* ATCC 14922 (GID: 1969.6) | soil (BV-BRC) |
| 102 | *Streptomyces coeruleorubidus* ATCC 13740 (GID: 116188.4) | soil (BV-BRC) |
| 103 | *Streptomyces davawensis* JCM 4913 (GID: 1214101.3) | soil (DOI: [10.1128/JB.01592-12](https://doi.org/10.1128/jb.01592-12)) |
| 104 | *Streptomyces griseorubiginosus* 3E-1 (GID: 67304.8) | soil (BV-BRC) |
| 105 | *Streptomyces griseorubiginosus*BTU6 (GID: 67304.10) | soil (BV-BRC) |
| 106 | *Streptomyces griseoviridis* F1-27 (GID: 45398.5) | NA (NA) |
| 107 | *Streptomyces hygroscopicus* XM201 (GID: 1912.5) | soil (BV-BRC) |
| 108 | *Streptomyces rapamycinicus* NRRL 5491 (GID: 1343740.8) | soil (DOI: [10.1128/genomeA.00581-13](https://doi.org/10.1128/genomea.00581-13)) |
| 109 | *Streptomyces scabiei* 87.22 (GID: 680198.5) | soil (BV-BRC) |
| 110 | *Streptomyces* sp. 11-1-2 (GID: 1851167.4) | soil (BV-BRC) |
| 111 | *Streptomyces* sp. CC0208 (GID: 2306165.3) | aquatic sediment (BV-BRC) |
| 112 | *Streptomyces* sp. CdTB01 (GID: 1725411.3) | soil (BV-BRC) |
| 113 | *Streptomyces* sp. Go-475 (GID: 2072505.3) | soil (BV-BRC) |
| 114 | *Streptomyces* sp. M2 (GID: 646637.3) | soil (BV-BRC) |
| 115 | *Streptomyces* sp. MK45 (GID: 2496836.3) | soil (BV-BRC) |
| 116 | *Streptomyces* sp. P3 (GID: 2135430.3) | soil (BV-BRC) |
| 117 | *Streptomyces* sp. RLB1-8Microscale soil grain (GID: 2594453.3) | soil (BV-BRC) |
| 118 | *Streptomyces* sp. RLB1-9Microscale soil grain (GID: 2594454.3) | soil (BV-BRC) |
| 119 | *Streptomyces* sp. RLB3-17Microscale soil grain (GID: 2594455.3) | soil (BV-BRC) |
| 120 | *Streptomyces* sp. RLB3-5Microscale soil grain (GID: 2594456.3) | soil (BV-BRC) |
| 121 | *Streptomyces* sp. RLB3-6Microscale soil grain (GID: 2594457.3) | soil (BV-BRC) |
| 122 | *Streptomyces* sp. S1A1-3Microscale soil grain (GID: 2594458.3) | soil (BV-BRC) |
| 123 | *Streptomyces* sp. S1A1-7Microscale soil grain (GID: 2594459.3) | soil (BV-BRC) |
| 124 | *Streptomyces* sp. S1A1-8Microscale soil grain (GID: 2594460.3) | soil (BV-BRC) |
| 125 | *Streptomyces* sp. S1D4-14Microscale soil grain (GID: 2594461.3) | soil (BV-BRC) |
| 126 | *Streptomyces* sp. S1D4-20Microscale soil grain (GID: 2594462.3) | soil (BV-BRC) |
| 127 | *Streptomyces* sp. S1D4-23Microscale soil grain (GID: 2594463.3) | soil (BV-BRC) |
| 128 | *Victivallales* *bacterium* CCUG 44730 (GID: 2094242.3) | GIT (BV-BRC) |
